# The bHLH Factors Extramacrochaetae and Daughterless Control Cell Cycle in *Drosophila* Imaginal Discs through the Transcriptional Regulation of the *cdc25* Phosphatase *string*

**DOI:** 10.1371/journal.pgen.1004233

**Published:** 2014-03-20

**Authors:** Irene Andrade-Zapata, Antonio Baonza

**Affiliations:** Centro de Biología Molecular Severo Ochoa (CSIC/UAM), Madrid, Spain; New York University, United States of America

## Abstract

One of the major issues in developmental biology is about having a better understanding of the mechanisms that regulate organ growth. Identifying these mechanisms is essential to understand the development processes that occur both in physiological and pathological conditions, such as cancer. The E protein family of basic helix-loop helix (bHLH) transcription factors, and their inhibitors the Id proteins, regulate cell proliferation in metazoans. This notion is further supported because the activity of these factors is frequently deregulated in cancerous cells. The E protein orthologue Daughterless (Da) and the Id orthologue Extramacrochaetae (Emc) are the only members of these classes of bHLH proteins in *Drosophila*. Although these factors are involved in controlling proliferation, the mechanism underlying this regulatory activity is poorly understood. Through a genetic analysis, we show that during the development of epithelial cells in the imaginal discs, the G2/M transition, and hence cell proliferation, is controlled by Emc via Da. In eukaryotic cells, the main activator of this transition is the Cdc25 phosphatase, *string*. Our genetic analyses reveal that the ectopic expression of *string* in cells with reduced levels of Emc or high levels of Da is sufficient to rescue the proliferative defects seen in these mutant cells. Moreover, we present evidence demonstrating a role of Da as a transcriptional repressor of *string*. Taken together, these findings define a mechanism through which Emc controls cell proliferation by regulating the activity of Da, which transcriptionally represses *string*.

## Author Summary

Precise control of cell proliferation is critical for normal development and tissue homeostasis. Members of the inhibitor of differentiation (Id) family of helix-loop-helix (HLH) proteins are key regulators that coordinate the balance between cell division and differentiation. These proteins exert this function in part by combining with ubiquitously expressed bHLH transcription factors (E proteins), preventing these transcription factors from forming functional hetero- or homodimeric DNA binding complexes. Deregulation of the activity of Id proteins frequently leads to tumour formation. The Daughterless (Da) and Extramacrochaetae (Emc) proteins are the only members of the E and Id families in *Drosophila*, yet their role in the control of cell proliferation has not been determined. In this study, we show that the elimination of *emc* or the ectopic expression of *da* arrests cells in the G2 phase of the cell cycle. Moreover, we demonstrate that *emc* controls cell proliferation via Da, which acts as a transcriptional repressor of the Cdc25 phosphatase *string*. These results provide an important insight into the mechanisms through which Id and E protein interactions control cell cycle progression and therefore how the disruption of the function of Id proteins can induce oncogenic transformation.

## Introduction

Cell proliferation is a critical event in organ formation and it is regulated by multiple cellular signals. During development, individual cells interpret these signals to determine whether to continue proliferating or to induce cell cycle arrest and initiate differentiation. The way that cells integrate these different signals to control the cell cycle machinery is a crucial issue that has received a great deal of attention [1]–[2] although many aspects of this process have yet to be elucidated.

The basic Helix-Loop-Helix (bHLH) family of transcription factors are key regulatory molecules that control multiple developmental processes in species from yeast to humans [3]. In metazoans, bHLH proteins orchestrate cell cycle control, cell lineage commitment and cell differentiation (reviewed in [4]). Dimerization of the HLH domain allows these proteins to form homo- or heterodimers, while their basic domain is responsible for their capacity to bind to DNA [5]–[6]. The bHLH proteins are classified into different categories according to their function, distribution and DNA-binding properties. Class II encompasses bHLH proteins that are expressed in tissue-specific patterns, including MyoD, Myogenin, NeuroD/BETA2, MASH, HAND and TAL. The activity of these factors is related to the acquisition of particular developmental fates or potentials. These tissue-specific HLH factors dimerize with ubiquitous bHLH proteins called E proteins, which are “class I” bHLH factors. Vertebrate class I bHLH proteins are encoded by differentially spliced transcripts from the E2A (E12, E47 and E2-5/ITF1 proteins), E2-2/ITF2 and HEB/HTF4 genes. The currently accepted model is that in these heteromers, the tissue-specific class II factors confer spatial and temporal specificity, whereas the ubiquitous class I factors drive DNA binding and transcriptional activation [7]–[9]. The basic domains of the dimers contact a DNA target site known as the E-box, which is responsible for transcriptional activation. As dimerization is essential for DNA binding and transcriptional activity *in vivo* [10], [4], proteins that prevent the formation of these complexes act as negative regulators. These negative regulators, which contain an HLH domain but lack a basic region, are members of the Inhibitor of Differentiation (Id) protein family, composed of Id1-4. These factors represent “class V” proteins and they bind to ubiquitous bHLH proteins to inhibit their activity [11]–[13].

Although Id proteins were discovered due to their ability to prevent cell differentiation, they play a broad range of biological roles during development and significantly contribute to tumour development [reviewed in 14]–[15]. In addition to the widely accepted function of E proteins as regulators of tissue specific gene expression, they are also believed to serve as cell cycle effectors. However, this latter function remains controversial due to the diversity of cell cycle-specific target genes identified for E proteins [16]–[19]. The functional redundancy between the members of these two families has also complicated the study of their influence on cell proliferation [20]–[21]. However, the problem of redundancy is eliminated in *Drosophila*, in which Daughterless (Da) and Extramacrochaetae (Emc) are the only representatives of classes I and V [22]–[24], thereby facilitating functional studies. The interaction between these proteins and with other bHLH proteins is a determinant of cell fate decisions in different developmental contexts [25]–[27]. Moreover, null mutations for *emc* cause severe defects in cell division, suggesting that *emc* may be necessary to maintain a proliferative state during organ development [28]–[29].

An evolutionary conserved regulatory network between Da and Emc, in which Da controls its own activity by enhancing *emc* expression, was described recently [30]. According to this model, Emc functions as a negative feedback regulator that prevents runaway self-stimulation of *da* expression. Thus, changes in the expression of *emc* by different extracellular signals modulate the levels of Da. Furthermore, because Emc can bind directly to Da, elimination of Emc promotes an increase in Da homodimers. Nevertheless, little is known about how Emc and Da control cell proliferation. In vertebrates, Id2 is thought to bind to the retinoblastoma tumour suppressor protein (pRB), abolishing its growth-suppressor activity [31]–[32], although no physical association between pRb and Emc has been detected in *Drosophila* [33]. To date, the role of *da* in regulating proliferation has not been studied.

In the present study, we investigated the control of cell proliferation by Da and Emc, and we found that both *emc* and *da* were required for the G2/M transition in the cell cycle. In eukaryotic cells, the main activator of mitosis is the (Cdc25) phosphatase String, which triggers the G2/M transition. We observed that defects in cell proliferation seen in *emc* mutant cells and *da* overexpressing cells were a result of reduced *string* expression. Thus, our results indicate that Da functions as a transcriptional repressor of *string*, thereby regulating G2/M transition.

The role of bHLHs in integrating signals and permitting cell fate acquisition has been studied previously, yet these proteins are also essential for cell proliferation and tissue growth. It is well established that Id factors regulate G1-S transition in vertebrate cells, and our data indicate that at least in *Drosophila*, E proteins can also control the G2/M transition in conjunction with Id factors. Thus, here we have described the first mechanism through which interacting class I and class V bHLHs intercede in this cell cycle transition.

## Results

### *emc* mutant cells are retained in the G2 phase of cell cycle

It was shown previously that cells completely lacking *emc* function do not survive in imaginal discs, suggesting a role for this gene in controlling cell proliferation and/or survival. In order to have a better understanding of the function of *emc* in the control of cell proliferation during discs development, we studied proliferation-related parameters in mitotic recombination clones of *emc* mutant cells generated using the mosaic analysis with repressible cell marker (MARCM) technique [34]. In these clones, GFP was positively expressed by all the mutant cells. Since clones of *emc* null cells do not survive, we used the strong hypomorphic *emc^1^* allele.

**Figure 1:**
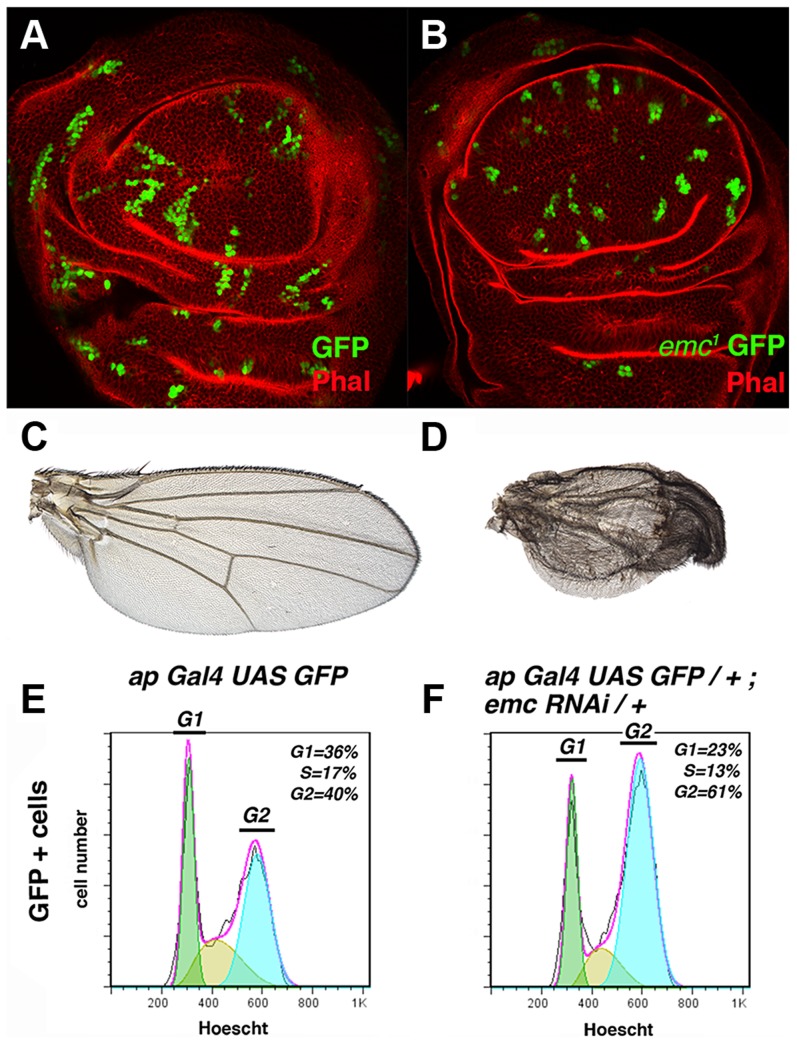
The absence of *emc* delays cell cycle progression in the G2 phase. (**A, B**) Third instar wing imaginal discs containing GFP-labelled (green) control (A) and *emc^1^* (B) mitotic recombinant clones. Phalloidin (Phal) staining is shown in red. The *emc^1^* mutant clones were always smaller than control clones (compare B to A). (**C, D**) Phenotype of *ap-Gal4 UAS-GFP/+* (C) and *ap-Gal4 UAS-GFP/+; UAS-emc^RNAi^/+* (D) adult wings. *ap-Gal4 UAS-GFP/+; UAS-emc^RNAi^/+* wings were smaller than the control wings, with vein fusions and extra bristles in the dorsal compartment (compare D to C). (**E, F**) FACS analysis of third instar wing imaginal discs from *ap-Gal4 UAS-GFP/+* (E) and *ap-Gal4 UAS-GFP/+; UAS-emc^RNAi^/+* (F) genotypes. Note the accumulation of cells in the G2 phase among the GFP + cells in the *emc^RNAi^* expressing discs (F, n = 4 independent experiments, p-value<0.05).

Clones of *emc^1^* mutant cells induced 60 h after egg laying (AEL) and analysed 48 h later were always smaller than the control clones (10.3±0.61 cells *vs* 23.8±1.2 in control clones, n = 40, p-value<0.001: Figure 1 A, B). In adult wings, these clones are elongated, they frequently appear to run along the veins and are much smaller than control clones. We also analysed the effects caused by *emc^1^* mutant clones when they have a proliferative advance upon surrounding cells using the *Minute (M)* technique. Clones of *M^+^* cells can out-compete the surrounding heterozygous *Minute^−^* cells, therefore these clones can grow into large wing territories. In adult wings, control *M^+^* clones tend to be restricted to the regions defined by veins (inter-vein regions), hence their sizes depend on the number of intervein regions that they occupy [28]. As expected, *emc^1^ M^+^* clones, induced 60 h AEL and analysed in adult wings, are larger than *emc^1^* clones, albeit smaller than control clones (1.3±0.7 intervein regions, n = 85 *vs* 2.2±1, in controls, n = 35) [35], [28], [29]. These mutant clones always caused a reduction in the size of the regions they occupy and can induce the fusion of adjacent veins, giving rise to the elimination of entire intervein regions [28] (Figure S1 A). These data indicate that despite the proliferative advantage provided by *M^+^*, cell proliferation was impaired in *emc^1^ M^+^* mutant cells.

The size reduction in *emc* mutant territories might have been due to increased cell death or cell cycle arrest or delay. To address whether the defects observed in these clones were caused by cell death, we blocked apoptosis in *emc^1^* mutant cells by simultaneously eliminating the proapoptotic *reaper (rpr), grim* and *hid* genes, using the deficiency *Df(3L)H99*. The deletion of these genes blocks apoptosis in *Drosophila* [36]. Clones of *emc^1^ Df(3L)H99 M^+^* mutant cells, induced 60 h AEL and analysed in adult wings, were indistinguishable from *emc^1^ M^+^* clones. The sizes of both *emc^1^ Df(3L)H99 M^+^* and *emc^1^ M^+^* clones were identical (1.3±0.63 inter-vein regions, n = 28 *vs* 1.3±0.7 in *emc^1^ M^+^*). In addition, as previously described for *emc^1^ M^+^* clones, the regions occupied by clones of *emc^1^ Df(3L)H99 M^+^* cells were heavily reduced in size, and they can induce the fusion of adjacent veins, causing the elimination of entire inter-vein regions (Figure S1 A).

Finally, in *emc^1^* clones we did not find apoptotic cells, as assayed by Caspase 3 staining in third instar wing discs (data not shown). All these data indicate that cell death was not the primary cause of the small size of *emc* mutant clones. We then investigated whether cell cycle progression was altered in *emc^1^* cells by determining cell doubling time [37]. The *emc^1^* clones induced at 60±12 h AEL and analysed 60±12 h later exhibited a prolonged cell doubling time that was delayed by 4.4 h compared to control clones (16.40 h in mutant *vs* 12 h in wt). This delay increased to 5 h when clones were analysed at 84±12 h after induction.

These results indicate that the rate of division of *emc* cells is slower than that of control cells, suggesting that the cell cycle is either blocked or delayed in these mutant cells. Based on these findings, we determined the proportion of *emc* mutant cells in the different stages of the cell cycle by Fluorescence-Activated Cell Sorting (FACS). To generate large *emc* mutant regions, we blocked *emc* activity by expressing a *UAS-emc^RNAi^* using the Gal4/UAS system. This *UAS-emc^RNAi^* was co-expressed with *UAS-GFP*, which allowed us to study the changes in cell cycle progression caused by the absence of *emc* in a defined cell population (GFP^+^ cells) and compare this to control cells in *ap-Gal4 UAS-GFP* discs. The adult wing phenotypes displayed by mutant flies after expression of the *UAS-emc^RNAi^* construct under the regulation of *ap-Gal4* were very similar to those produced by large *emc M^+^* mutant clones [28]. Thus, *UAS*-*emc^RNAi^ ap-Gal4* wings exhibited wing vein fusion that resulted in the elimination of inter-vein regions (Figure 1 D). Accordingly, we detected a strong reduction in the number of dividing cells in the dorsal compartment of these discs (35% reduction in the number of dividing cells compared to control discs, revealed by staining for the phospho-histone 3 mitotic marker (PH3)).

The FACS analysis revealed that a greater proportion of the *emc* mutant cells were in the G2 phase compared to the control cells in *ap-Gal4 UAS-GFP* discs (40% and 61% in control and mutant cells, respectively, n = 4, p-value<0.05: Figure 1 E, F). Similar results were obtained when the *UAS-emc^RNAi^* was expressed in the entire wing pouch under the regulation of the *nubbin*-*Gal4* driver (data not shown). Overall, these data indicate that although *emc* mutant cells can divide, they persist in the G2 phase for longer than control cells.

### The ectopic expression of Da retains cell cycle progression in the G2 phase

It was recently demonstrated that most of the effects of eliminating *emc* function are caused by increased Da expression in these mutant cells. Indeed, eliminating *da* in *emc* mutant cells was sufficient to rescue the poor viability of these cells in the Drosophila eye disc [30]. This hypothesis was confirmed in the imaginal wing discs in which clones of *emc^AP6^* cells barely survived, whereas *da emc* double mutant clones achieved a relatively normal size (Figure S2 A–C).

**Figure 2:**
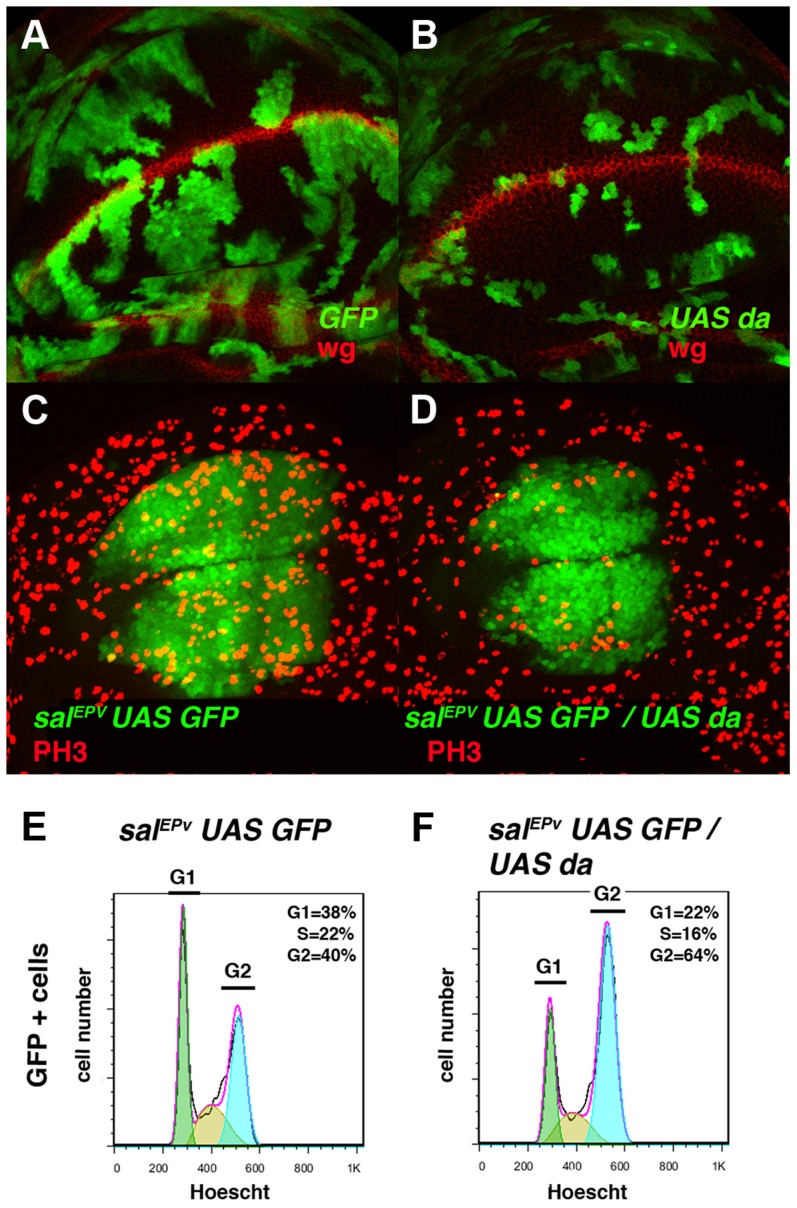
Overexpression of *da* mimics the phenotype induced by the absence of *emc*. (**A, B**) Third instar wing imaginal discs with Flip-out control (A) and *UAS-da* (B) clones labelled with GFP (green) and stained for Wingless (Wg, in red). Clones of *da*-expressing cells were smaller than the control clones (compare B to A). (**C, D**) Third instar *sal^EPv^-Gal4 UAS-GFP/+* (C) and *sal^EPv^-Gal4 UAS-GFP/UAS-da* (D) wing imaginal discs stained for the mitotic marker phospho-histone-3 (PH3). This marker was strongly diminished in the *sal^EPv^* expression domain (in green) of *da*-expressing discs. Accordingly, this region was smaller in mutant versus control discs (compare D with C). (**E, F**) FACS analysis of third instar wing imaginal discs of the *sal^EPv^-Gal4 UAS-GFP/+* (E) and *sal^EPv^-Gal4 UAS-GFP/UAS-da* (F) genotypes. Note the accumulation of GFP ^+^ cells from the *UAS-da* expressing discs in the G2 phase (F, n = 3 independent experiments, p-value<0.05).

**Figure 3:**
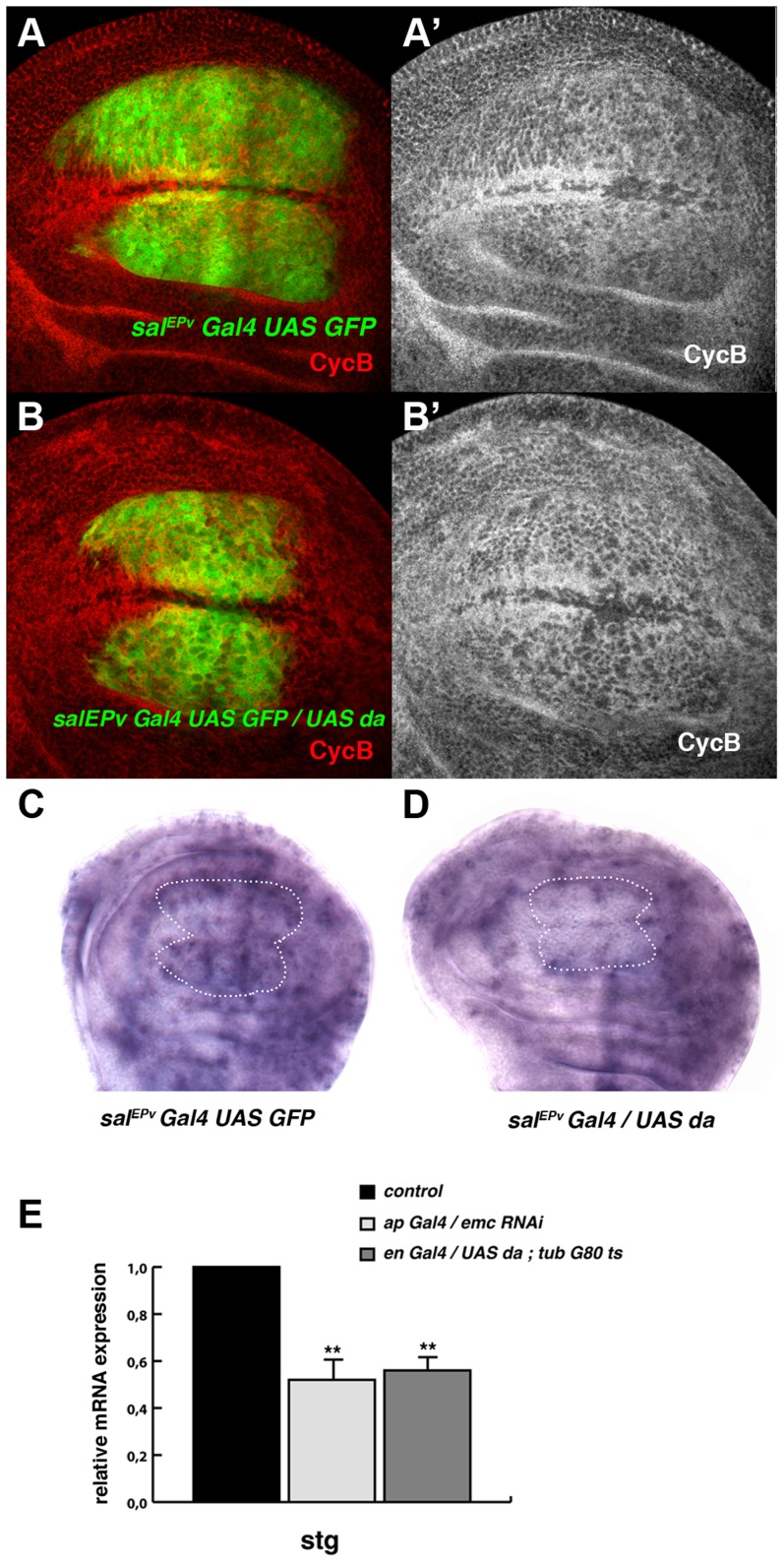
Da regulates the expression of *string*. (**A, B**) Expression of Cyclin B (CycB) in *sal^EPv^-Gal4 UAS-GFP/+* (A, A′) and *sal^EPv^-Gal4 UAS-GFP/UAS-da* (B, B′) third instar imaginal wing discs revealed with anti-CycB (red in A and B and grey in A′–B′). The expression of CycB in *sal^EPv^-Gal4 UAS-GFP/UAS-da* discs was comparable to that of the control discs (compare B′ to A′). (**C, D**) *In situ* hybridization to *string* mRNA in *sal^EPv^-Gal4 UAS-GFP/+* control (C) and *sal^EPv^-Gal4 UAS-GFP/UAS-da* (D) third instar wing imaginal discs. The *sal^EPv^-Gal4* presumptive area is marked with a white dotted line. Note that the *stg* expression was strongly reduced when *da* was overexpressed (D). (**E**) Quantitative Real-Time PCR of cDNA from imaginal wing discs of genotypes *WT*, *ap-Gal4/+; UAS-emc^RNAi^* and *en-Gal4/UAS-da; tubG80^ts^/+.* *emc* reduction and *da* overexpression caused a significant reduction in *stg* mRNA when *da* was overexpressed (n = 3 independent experiments, **p<0.01 *vs WT)*.

**Figure 4:**
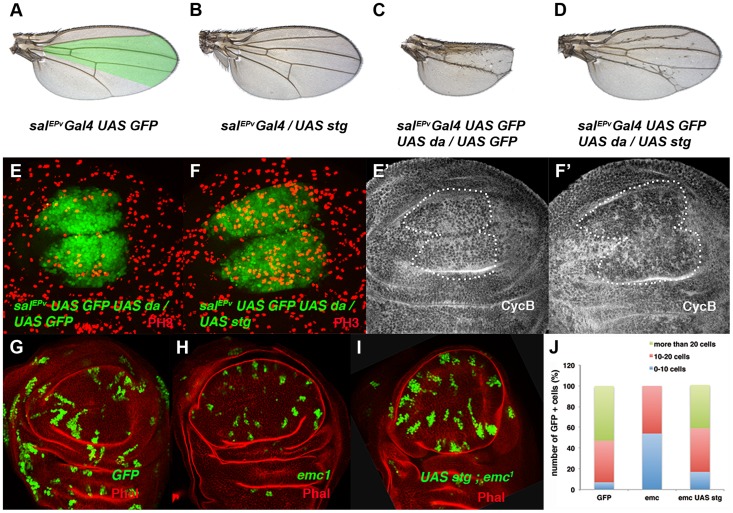
The ectopic expression of *string* is sufficient to restore the mitotic defects induced by the elimination of *emc* or the ectopic expression of *da*. (**A–D**) Adult wings of *sal^EPv^-Gal4 UAS-GFP/+* (A), *sal^EPv^-Gal4 UAS-GFP/UAS-stg* (B), *sal^EPv^-Gal4 UAS-GFP UAS-da/UAS-GFP* (C), and *sal^EPv^-Gal4 UAS-GFP UAS-da/UAS-stg* (D). Note that the defects in wing size observed following *da* overexpression were reverted by *stg* overexpression (compare C to D). (**E, F**) Phospho-histone-3 (PH3) staining (in red) in *sal^EPv^-Gal4 UAS-GFP UAS-da/UAS-GFP* (E) and *sal^EPv^-Gal4 UAS-GFP UAS-da/UAS-stg* (F) third instar wing imaginal discs. (E′, F′) CycB expression was reduced when *stg* was overexpressed (alone or in combination with *UAS-da*), due to the rapid G2/M transition of these cells. (**G, H, I**) Third instar wing imaginal discs containing control (G), *emc^1^* (H) and *UAS-stg*; *emc^1^* (I) clones were, positively labelled by GFP (in green) and stained with Phalloidin (Phal, red). The smaller size of the *emc^1^* mutant clones was reverted by overexpressing *stg* (compare H to I). (**J**) Quantitative analysis of the size of *control*, *emc^1^* and *UAS-stg*; *emc^1^* clones. Note that the *emc^1^* mutant clones were always smaller than 20 cells, whereas *UAS-stg*; *emc^1^* clones were of a similar size to the control clones (n = 40 clones per group).

We examined whether the ectopic expression of *da* mimicked the effects of *emc* depletion on cell cycle progression. To this end we generated clones of *da*-expressing cells using the Gal4/UAS system combined with the Flip-out technique (see Matherials & Methods). As seen in clones of *emc* mutant cells, clones of cells ectopically expressing *da* were very small compared to controls (Figure 2 A, B). Accordingly, overexpression of *da* in the central region of the wing blade using *sal^EPv^-Gal4* (*sal^EPv^-Gal4 UAS-GFP/UAS*-*da*) reduced the size of this region in third instar wing discs (Figure 2 C, D, and Figure 3) and adult flies (Figure 4 C). In these adult wings, several veins were fused, provoking the elimination of inter-vein regions (compare Figures 4 C with 4 A). This effect was correlated with a reduction in the mitotic index (PH3-positive cells/size of *sal^EPv^-GFP* region in pixel) in the domain of *sal^EPv^* expression (3.13±0.18 mitotic index in control *vs* 1.08±0.09 in mutant discs, n = 20, p-value<0.001: Figure 2 C, D).

In concordance with the results found when *emc* was reduced in the discs, a FACS analysis in *sal^EPv^-Gal4 UAS-GFP/UAS*-*da* discs revealed an increase in the proportion of cells in the G2 phase of the cell cycle in cells overexpressing *da* (39.79% in control *sal^EPv^-Gal4 UAS-GFP*/+ *vs* 64.49% in *sal^EP^ -Gal4/UAS-da* discs, n = 3, p-value<0.05: Figure 2 E, F). Consistently with the model in which Emc titrates Da, we observed that the overexpression of *emc* partially suppressed the cell proliferation defects caused by the overexpression of *da*. Thus, we found that when *UAS-da* and *UAS-emc* were simultaneously overexpressed under the control of *sal^EPv^-Gal4 UAS-GFP*, the density of PH3 positive cells in the domain of expression of *sal^EPv^* was significantly higher than in wing discs in which only *UAS*-*da* was overexpressed (mitotic index of *sal^EPv^-Gal4/UAS-da; UAS emc* discs 1.51±0.32 compared with 1±0.14 when only *UAS-da* was overexpressed; n = 8, p-value = 0.004: Figure S2 H–K). Consequently, the size reduction of the adult wings caused by the overexpression of *da* was strongly suppressed in these doubly mutant wings (Figure S2 D–G). We then studied whether cell death was causing the size reduction phenotype produced by *da* overexpression. To this end, we co-expressed the apoptosis inhibitor Diap I in flies that simultaneously overexpressed *da*. The overexpression of *diap* suppressed neither the reduced wing size phenotype caused by the ectopic expression of *UAS-da* nor the deficiency of mitotic cells observed in wing discs than solely overexpressed *UAS-da* (mitotic index of *sal^EPv^-Gal4/UAS-da; UAS-diap* discs 0.98±0.21 *vs* 1±0.14 for *UAS-da* discs; n = 10; Figure S1 B–G). Moreover, we detected no apoptotic cells in wing discs containing clones of *da*-expressing cells (data not shown). As previously described in mutant condition for *emc*, these data support the idea that cell death was not the primary cause of the defect found when *da* was overexpressed.

Taken together, our data support the hypothesis that in *emc* mutant cells the increased expression of *da* reduces the rate of cell division, arresting cell cycle progression in the G2 phase or slowing G2/M transition.

### Emc and Da regulate the expression of *string*

Based on the accumulation of cells in the G2 phase caused by the downregulation of *emc* or the overexpression of *da*, we examined the expression of regulators of the G2/M transition in cells overexpressing *da*. The CycB-CDK1 complex is essential for the transition from the G2 to M phase and this complex is dephosphorylated, and thereby activated, by the universal activator of mitosis in eukaryotic cells, the Cdc25 phosphatase String. The transcriptional activation of *string* triggers mitosis. We found that the expression of CycB in *sal^EPv^-Gal4 UAS-GFP/UAS*-*da* was comparable to that which is seen in control discs (Figure 3 A, B) and we therefore examined *string* mRNA expression in these discs by *in situ* hybridization. Interestingly, the expression of *string* mRNA was strongly reduced in the central region of *sal^EPv^-Gal4 UAS-GFP/UAS*-*da* wing discs compared to control discs (Figure 3 C, D).

We further analysed the regulation of *stg* by *da* through quantitative Real-Time PCR (qRT-PCR). Using the *Gal4*/*Gal 80* ^Ts^ system, we overexpressed *da* in third instar *engrailed*-*Gal4 (en-Gal4)/UAS*-*da; tub*-*Gal80^ts^* wing discs and we quantified the total amount of *stg* mRNA 48 h after the induction of *da*. The expression of *stg* mRNA was reduced in mutant discs compared to control discs. Similar results were obtained when *emc* function was blocked by overexpressing an *UAS-emc^RNAi^* throughout development under the regulation of *ap-Gal4* (Figure 3 E). In contrast, the levels of expression of mRNA of *cycB*, which is also specifically expressed in the G2 phase of the cell cycle, were not affected after the overexpression of *da* (data not shown). This result suggests that the overexpression of *da* was not indiscriminately affecting the expression of all the genes required for the G2/M transition.

The downregulation of *string* caused by the ectopic expression of *da* could account for the accumulation of cells in the G2 phase. Alternatively, *da* may repress other factors that regulate G2/M transition. To investigate these possibilities we co-expressed *da* and *string* under the regulation of *sal^EPv^-Gal4*. As mentioned above, overexpression of *da* in the domain of *sal^EPv^* expression reduced the size of this region in adult wings (compare Figures 4 A and 4 C). Strikingly, this reduction was almost completely suppressed by *UAS-string* co-expression (compare Figure 4 D with 4 C). Accordingly, in third instar wing discs co-expressing *UAS-da* and *UAS-string*, PH3 staining revealed the restoration of cell division (Mitotic index: *sal^EPv^-Gal4 UAS-GFP*: 3.01±0.04; *sal^EPv^-Gal4 UAS-GFP/UAS*-*da*: 1.71±0.04; *sal^EPv^-Gal4 UAS-GFP/UAS-stg*: 7.04±0.17; *sal^EPv^-Gal4 UAS-GFP-UAS*-*da/UAS-stg*: 4.43±0.17) (compare Figure 4 F with 4 E). Since adult wings overexpressing *da* and *stg* were slightly smaller than the *sal^EPv^-Gal4* controls, we cannot rule out the possibility that Da might be required for the regulation of other cell cycle regulators in addition to *string*. We observed that CycB expression was downregulated, indicating that G2/M transition was accelerated, as occurred when *string* alone was overexpressed (Figure 4 E′–F′ and data not shown). Interestingly, extra-bristle differentiation continued in the rescued adult wings, indicating that the influence of *da* on mitosis and differentiation is distinct (Figure 4 C, D). Similar results were obtained when *string* was overexpressed in *emc* mutant cells (Figure 4 G–I). Thus, the average number of cells that formed *UAS-string emc^1^* double mutant clones increased to 20.4±1.28 (23.8±1.2 in control clones) from 10.3±0.61 in *emc^1^* clones (Figure 4 J). Moreover, clones formed by more than 20 cells, which were absent in *emc^1^* discs, reappeared in the *UAS-string emc^1^* double mutant clones (Figure 4 J), and the extended doubling time of *emc^1^* clones was restored by *string* overexpression (13.88 h in *UAS-string emc^1^* clones *vs* 13.08 h in control clones). Finally, the proliferative defect observed following *emc* depletion by *UAS-emc^RNAi^* was also suppressed by *string* overexpression (Figure S3).

These results indicate that loss of *string* expression is the main cell cycle defect caused by the overexpression of *da* or the absence of *emc*. Although our results demonstrate that *da* plays an important role in repressing the transcription of *string*, the downregulation of *da* was insufficient to increase *string* expression, as revealed by *in situ* hybridization and quantitative Real-Time PCR of *stg* mRNA in *ap-Gal4 UAS-da-^RNAi^* wing discs (Figure S4). Different alternatives can explain these apparently contradictory results (see Discussion).

### Da binds to the *string* promoter region

Our results are consistent with the view that *string* is repressed by Da. Considering that Da is a transcriptional factor, the simplest molecular mechanism to explain this regulatory effect would be that Da acts directly on the *stg* promoter. To test this idea, we scanned the promoter region of *string* for putative Da-binding sites, given that Da binds to the E box consensus sequence CAC/GCTG. The transcription of *string* in *Drosophila* is regulated by a large 5′ region (>40 kb) that contains many regulatory elements [38], including multiple putative Da binding sites that we could identify in the *string* promoter.

**Figure 5:**
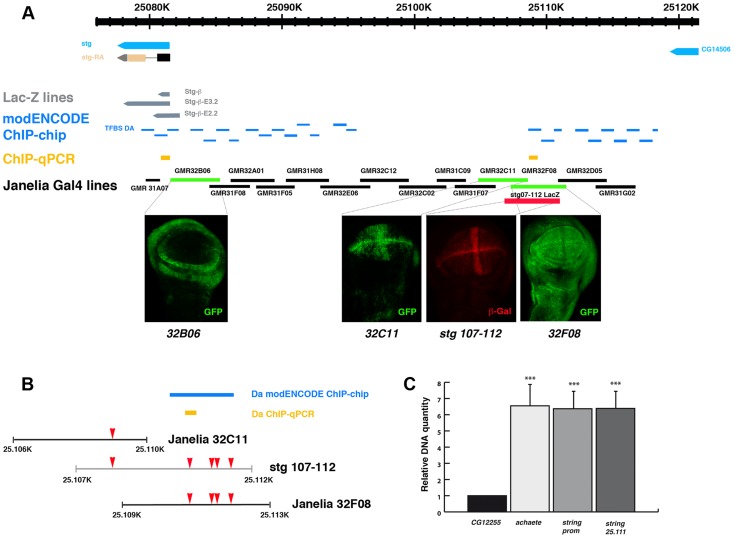
Da can bind *string* regulatory region. (**A**) Scheme representing *string* regulatory region. The LacZ lines generated by B. Edgar lab *stg-ß, stg_ ß-E3.2* and *stg_ ß-E2.2* are indicated in grey. The results obtained by the modENCODE ChIP project for Daughterless transcription factor are indicated in blue. Every blue line indicates a fragment of DNA that was immunoprecipitated with an anti-Da antibody. The orange lines marked as “ChIP-qPCR” represent the fragments studied by us in our ChIP experiment. The Gal4 lines generated by the Janelia Farm covering the *stg* regulatory region are shown in black. We highlighted the lines with expression in the imaginal wing disc (marked by GFP expression, in green in the discs), which are the *GMR_32B06*, *GMR_32C11* line and the *GMR_32F08* line. The *stg-107–112-LacZ* line, generated in F. Casares lab was also included, and a disc stained with anti- ß-Gal (in red) was shown. Note the similarity in the expression of this line with the *GMR_32C11* line. (**B**) Detail of a fragment of the *stg* regulatory region contained by the Janelia *GMR_32C11* and *GMR_32F08* lines and the *stg-107–112-LacZ* line. All Da putative binding sites present in this region are indicated with a red arrowhead. We also show the fragment found in the modENCODE project as a target for Da binding (blue line) and the fragment found by us (orange line). (**C**) Graphical representation of our ChIP-qPCR experiment. Each bar represents the relative DNA quantity immunoprecipitated with the anti-Da antibody. The gene *CG12255* was studied as negative control (see Matherials & Methods). *achaete* promoter was used as an internal positive control for the experiment. The *string* fragments indicated in (A) and (B) with an orange line were also represented. Note that Da binding to the *stg* promoter was very similar to that which was observed for the *achaete* promoter.

To further investigate whether these binding sites were involved in the regulation of *stg* expression, we first examined the *stg* promoter to map transcriptional control elements required to regulate its expression during disc development. To this end we analysed the LacZ expression of a collection of *string-LacZ* reporters containing promoter fragments (between −1 kb and +5 kb) thought to regulate *string* expression in wing discs [38] (Figure 5). None of these reporters were expressed in third instar imaginal wing disc (data not shown), suggesting that the regulatory regions required to activate *string* expression in the wing discs are located in other regions of the promoter. In the light of these data, we decided to extend our study to the rest of the promoter region. We subsequently examined the pattern of expression of a collection of *Gal4* lines that were under the control of defined sequence fragments from flanking non-coding or intronic regions of different genes throughout Drosophila genome (GMR_Brain_exp_1 or Rubin Gal4 lines, Janelia Farm). We selected the lines that contained fragments of the *stg* promoter (Figure 5). When these lines were combined with *UAS-GFP*, we found that the *GMR_32B06*, *GMR_32C11* and *GMR_32F08* lines expressed *UAS-GFP* in third instar imaginal wing disc. The *GMR_32F08* line drove the expression of *UAS-GFP* in all the cells of the wing discs, whereas the *GMR_32B06* and *GMR_32C11* lines expressed *UAS-GFP* in a defined pattern. Thus, the *GMR_32B06* line drove *UAS-GFP* expression in the region corresponding to the putative hinge of the wing disc, while in the *GMR_32C11* line the expression of *UAS-GFP* was restricted to wing blade region, where it was expressed at higher levels in the putative wing margin and in a band of cells at the anterior/posterior compartment boundary, in a manner similar to the expression of *string* in third instar wing discs (Figure 5 and Figure S5).

**Figure 6:**
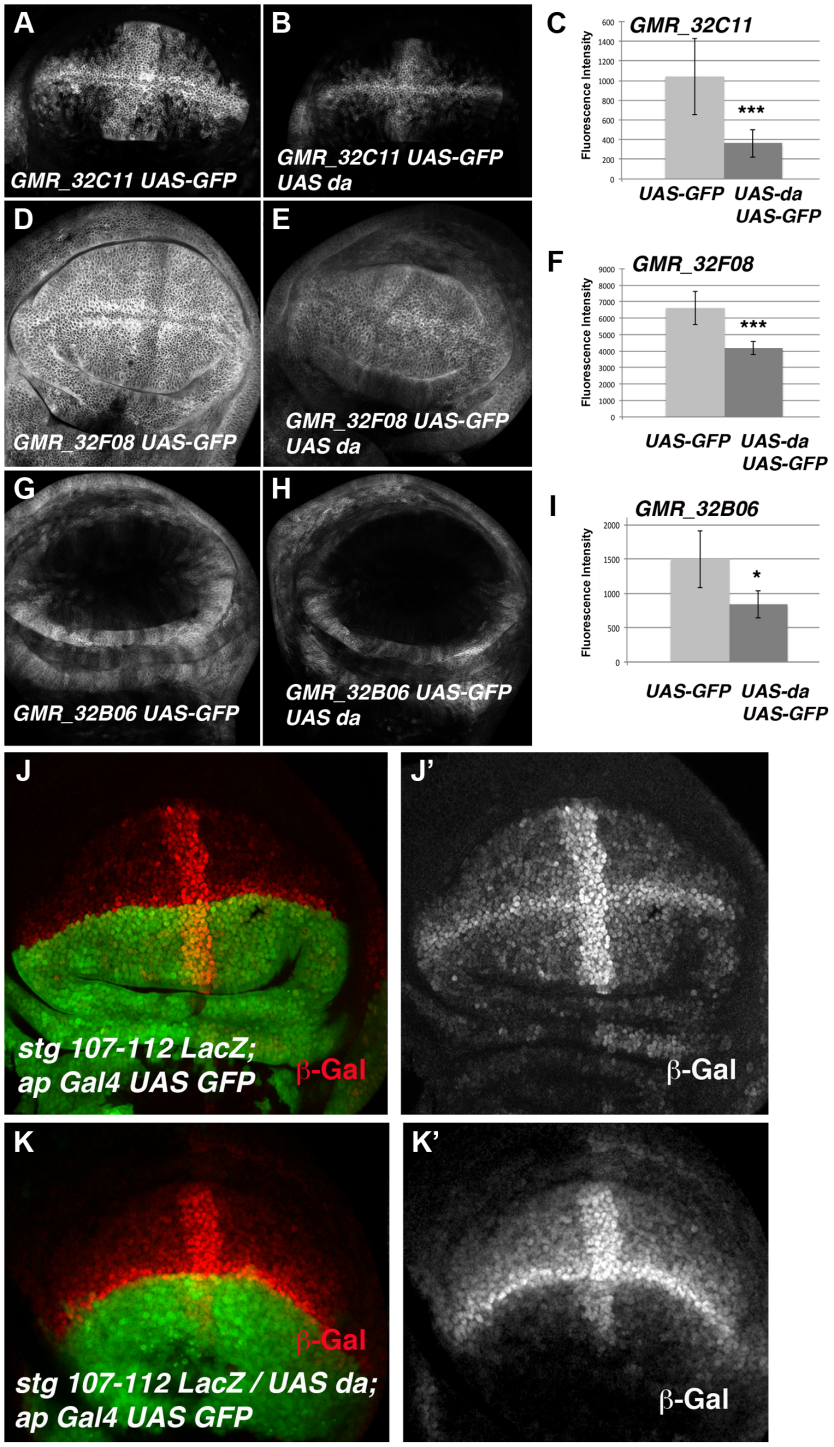
The over-expression of *da* down-regulates the expression of different *stg*-reporters. (**A, B, D, E, G** and **H**) Third instar larval wing discs showing the expression of *UAS-mCD8-GFP* (in grey) driven by the Janelia Gal4 lines *GMR_32C11* (A), *GMR_32F08* (D) and *GMR_32B06* (G) in control discs or in discs that over-express *UAS-da, GMR_32C11*-*Gal4 UAS-mCD8-GFP/UAS-da* (B), *GMR_32F06*-*Gal4 UAS-mCD8-GFP/UAS-da* (E) and *GMR_32B06*-*Gal4 UAS-mCD8-GFP/UAS-da* (H). (**C, F**, and **I**) Bar charts show the average levels of *mCD8-GFP* expression in control (*UAS-GFP*) and discs over-expresing *UAS-da* (*UAS-GFP UAS-da*) driven by *GMR_32C11*, *GMR_32F08* and *GMR_32B06* lines. For each experiments at least 9 wing discs were quantified. (B) The over-expression of *UAS*-*da* under the control of *GMR_32C11 Gal4* caused the down-regulation of the levels of *UAS-GFP* expressed in the wing pouch. The band of cells expressing this reporter along the A/P boundary was also reduced (compared B to A, *** p-value = 0.001). (E) The over-expression of *UAS-da* under the regulation of *GMR_32F08 Gal4* line reduced the levels of expression of GFP throughout the wing disc, compared with the control in D (*** p-value<0.001). (H) The expression of *UAS-GFP* reporter was also reduced in the proximal region of the wings discs when *UAS-da* was over-expressed with the *GMR_32B06* line (compared H to control G, * p-value<0.05). (**J-K**) Third instar larval wing discs stained with anti-ß-Gal to reveal the pattern of expression of the *stg-107–112-LacZ* reporter (in red in J, K and in grey in J′ K′). In *stg-107–112-LacZ*/*UAS*-*da*; *ap-Gal4* wing discs (K–K′) the expression of this reporter was strongly reduced throughout the dorsal compartment (marked by the expression of *UAS-GFP* in green), compared to control discs J–J′. Note the strong reduction of the expression of this reporter in the dorsal band of cells along the A/P boundary.

To determine whether the regulatory regions contained in the *GMR_32B06, GMR_32C11* and *GMR_32F08 Gal4* lines were regulated by Da, we analysed the expression of *UAS-GFP* driven by these lines when *da* was overexpressed. To this end, we crossed these *Gal4* lines combined with *UAS-GFP* by *UAS-da* and studied the levels of GFP in third instar wing discs. Transcriptional reporter activity was established by quantifying GFP levels in control third instar discs (no *UAS-da*) and comparing them to the levels of GFP observed in discs that also overexpressed *UAS-da* (see Matherials & Methods). We found that increasing *da* levels in the wing disc reduced the levels of expression of GFP in the three lines analysed (Figure 6 A–I). Thus, when *da* was overexpressed under the control of *GMR_32B06* and *GMR_32C11* lines, both the number of cells expressing GFP and the levels of expression of this protein were reduced (Figure 6 A–C and G–I). In wing discs in which *UAS-GFP* and *UAS-da* were under the control of the *GMR_32F08* line, we found that although *UAS-GFP* was expressed in all the cells of the discs, its levels of expression were strongly reduced compared to the control disc (Figure 6 D–F).

To further investigate the function of *da* regulating the transcriptional activity of *stg* promoter in the wing blade region, we used a *Lac-Z* reporter construct (*stg-107–112 LacZ*, kindly provided by C.S. Lopes. and F. Casares) containing almost the entire fragment presented in the *GMR_32C11* and *GMR_32F08 Gal4* lines (Figure 5 B). This reporter was sufficient to drive ß-Gal expression in most of the wing blade cells, with higher levels in the cells of the wing margin and in a band of cells in the A/P boundary, in a pattern resembling the one obtained with the line *GMR_32C11* (Figure 5 A and 6). Interestingly, the expression of this reporter was very similar to the pattern of expression of *emc* (Figure S5). Consistent with our previous results, we found that ß-Gal expression driven by *stg-107–112 LacZ* was strongly reduced in the dorsal compartment of third instar wing discs, in which *da* was overexpressed under the control of *ap-Gal4* (*ap-Gal4 UAS-GFP/UAS*-*da*) (Figure 6 J–K).

The expression activity of this reporter was not affected when *da* was downregulated. Thus, when the function of *da* was reduced in third instar *en-Gal4 UAS-GFP/stg-107–112 LacZ; UAS-da^RNAi^* wing discs, we found that ß-Gal expression driven by *107–112 LacZ* reporter in the anterior control compartment was comparable to the expression of this reporter in the posterior compartment (Figure S4 F–F′). This result is consistent with the observation that the reduction in *da* function was not sufficient to increase *string* mRNA expression (see above).

Taken together, these data indicate that Da can transcriptionally control different regulatory elements present in the promoter region of *stg*.

As was mentioned previously, we identified multiple putative Da binding sites throughout *string* promoter. Interestingly, a map of the *Drosophila melanogaster* regulatory genome was recently produced on the basis of different chromatin immunoprecipitation data sets, including thirty-eight site-specific transcription factors (modENCODE cis-regulatory annotation project) [39]. One of the transcriptional factors used in this work was Da. The data presented in this study indicate that Da binds *in vivo* to multiple regions in the promoter region of *stg* (Figure 5 A, B). We found five putative Da binding sites in the regulatory region that we have identified as being involved in the regulation of *string* expression in wing discs (between nucleotides 25.106 and 25.113) (Figure 5 B). The modENCODE cis-regulatory annotation indicates that Da binds to this region *in vivo* (Figure 5 B). We conducted an additional ChIP assay using an antibody specific against Da. To quantify the amount of precipitated DNA, quantitative Real-Time PCR was preformed after the ChIP, using primers for *stg*, *achaete* and *CG12255* promoters. We used *achaete* as a positive control, as it has been previously reported that Da binds to this promoter, and *CG12255* as a negative control, as it does not contain putative Da binding sites. For the *stg* promoter we analysed two regions, one located between 25.081.039–25.081.121 (Chr3R) (between nucleotides −976 and −205 upstream of the first transcriptional initiation site), and the other between nucleotides 25.111.328–25.111.401 (Chr3R) (29 Kb from the first transcriptional initiation site), including one putative Da binding site that lies in the regulatory region that we have identified to be required to control *string* expression during wing disc development (Figure 5 B) (see Matherials & Methods). We found that compared to the *CG12255*, DNA from both *stg* regulatory regions was enriched at the same levels as the *achaetae* promoter (Figure 5 C). This result confirms the data reported by modENCODE, and indicates that Da binds to the regulatory regions that we have identified as being involved in the regulation of *string* expression in wing discs.

### Tissue-specific bHLH proteins of the E(spl) and Ac/Sc complexes do not mediate the effects of Da on cell proliferation

It has been proposed that heterodimers formed between Da and other bHLH factors promote the transcriptional activation of different target genes. Since our results suggest that Da behaves as a transcriptional repressor of *string*, this effect could be mediated by other bHLH factors, as Da could partner any number of bHLH proteins expressed during disc development. Indeed, it has been proposed that the heterodimers formed by Da and the bHLH protein Twist result in the repression of somatic myogenesis [40].

**Figure 7:**
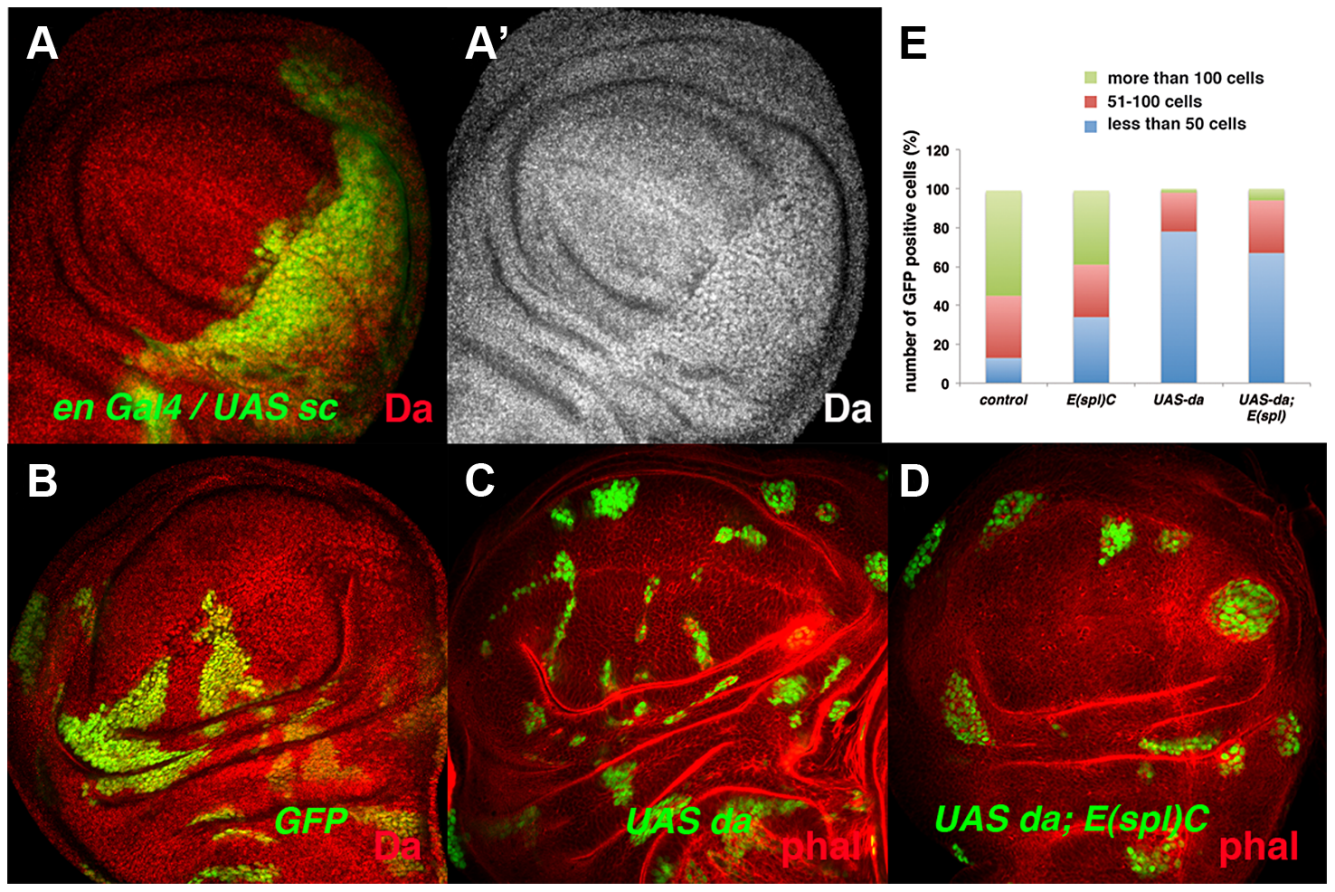
The ectopic expression of *da* alters the parameters of cell proliferation in the absence of the *E(spl)C* genes. (**A**) Expression of Da (red in A and grey in A′) in *en-Gal4 UAS-GFP/UAS-sc* third instar imaginal wing discs. The expression of Da was upregulated by the overexpression of *sc* (compare posterior GFP+ cells to anterior cells). (**B–D**) Third instar wing imaginal discs containing GFP^+^ (green) control (B), *UAS-da* (C) and *UAS-da; E(spl)^b32.2^* (D) mitotic recombinant clones. The discs were also stained with anti-Da (red) (B) and Phalloidin (Phal, red: C, D). *UAS da* and *UAS da; E(spl)C* clones were always smaller than control clones (compare C and D to B). (**E**) Quantification of the size of control, *E(spl)^b32.2^*, *UAS-da* and *UAS- da; E(spl)^b32.2^* clones (n = 37, 29, 91 and 63 analysed clones, respectively). Note that most control and *E(spl)C* clones contained more than 100 cells (40%–50% of clones), whereas only around 6% of the *UAS-da* and *UAS-da; E(spl)^b32.2^* reached this size.

During wing disc development, the tissue-specific bHLH factors Achaete (*ac*) and Scute (*sc*) are expressed in the presumptive wing margin. These proneural genes prefigure the pattern of sensory elements and they are required to define the sensory organs of the wing discs. At this stage, the cells located in the presumptive wing margin are arrested in the G2 phase of the cell cycle and they establish the so-called zone of non-proliferative cells (ZNC) [41]–[42]. Ectopic expression of *sc* in the ZNC results in the loss of *stg* expression [43], suggesting that these proneural genes might interact with Da and mediate its repressor activity. However, *ac* and *sc* depletion does not reverse the proliferative defects observed in *emc* mutant cells, as clones of *emc Df(sc)^19^* cells are similar to *emc* mutant clones [35]. The capacity of Sc to repress *stg* expression in the ZNC could be explained if Sc directly or indirectly alters Da levels. To test this possibility, we overexpressed *sc* in third instar *en*-*Gal4/UAS*-*sc* wing discs and analysed the expression of Da. We found that in these discs the expression of Da was strongly enhanced in the posterior compartment compared to the anterior compartment (Figure 7 A, A′). Taken together, these data indicate that Da regulates cell proliferation independently of Sc and Ac. They also suggest that the capacity of Sc to repress *stg* expression, and hence proliferation in the ZNC, is due to its ability to modulate the levels of Da, although further studies are required to confirm the mechanistic details of this regulation.

Other bHLH proteins dynamically expressed in epithelial cells are encoded by the seven genes that comprise the *Enhancer-of-split complex (E(spl)C)* [44]–[46]. During wing disc development, the *E(spl)* genes are required to single out sensory organ precursors and vein patterning, and they at least partially mediate the role of Notch signalling in the regulation of cell proliferation [47]–[48]. To study whether members of the *E(spl)* complex mediate the function of Da in the control of cell proliferation, we examined the phenotype of clones for a deficiency of *E(spl) (E(spl)^b32.2^*) that simultaneously overexpressed *UAS*-*da*. These clones were much smaller than control clones and similar in size to the clones of *da*-expressing cells (Figure 7 B–E). These results indicate that high levels of Da expression can alter cell proliferation even in the absence of *E(spl)* genes.

**Figure 8:**
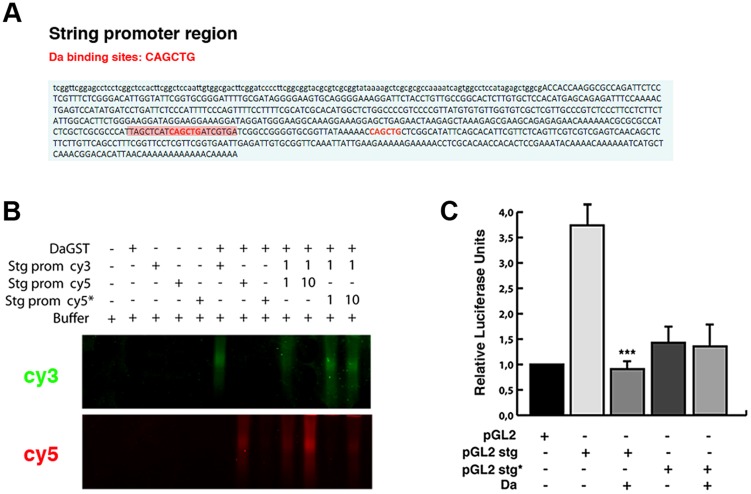
Da directly binds to the *string* promoter region and acts as a transcriptional repressor. (**A**) Scheme of the 0.7 Kb region of the *stg* promoter. The two E-boxes with the canonical Da binding sites CAG/CCTG contained in this region are written in red. The highlighted red region of 21 nucleotides corresponds to the probe used to perform the band shift experiments. (**B**) Band shift assay showing the physical binding of a Da-GST fusion protein to the *stg* promoter region. The DNA binding properties of Da were tested using cyc5 stg-prom (red), cyc3 stg-prom (green) and cyc5 stg-prom* (red) probes. The putative Da binding sites were mutated in the latter probe. Da bound to the probe containing the E-box CAGCTG but not to the mutated probe. This binding was specific, as oligonucleotides with a mutated binding site did not compete for binding. The gel was cut to show only the specific band formed by DNA-protein complexes. (**C**) Luciferase reporter assay of S2 cells. The 0.7 Kb region of the *stg* promoter shown in A was cloned into a pGL2 vector containing a minimal promoter (HS43), a construct that induced the activation of luciferase. The presence of the *stg* regulatory region (pGL2 stg) increased the basal activity of the pGL2 vector, which was strongly repressed by overexpression of Da (***p<0.001 *vs* pGL2 stg). When Da binding sites were ablated from the promoter (pGL2 stg*), no activation of the luciferase activity was observed.

To further investigate whether the ectopic expression of Da alone is sufficient to repress *string* expression, we studied the activity of a *string* reporter in S2 cells, which do not express most of the tissue-specific bHLH class II proteins (FlyBase). We analysed the activity of the *luciferase* gene under the regulation of a region of 0.7 Kb of the *stg* promoter (from −976 bp to −205 bp, 0 represents the first transcriptional initiation site). Although none of the *Lac-Z* reporters containing this region was sufficient to express ß-Gal at detectable levels in third instar imaginal (see above), probably because this region lacks the regulatory regions that we have identified to be necessary to activate *string* to its normal levels in the wing discs, it has previously been proposed that this fragment was required to regulate *string* activity during disc growth [38]. Thus, *string* transgenes possessing this 0.7 Kb region were sufficient to strongly rescue cell proliferation in *stg* mutant cells during disc development [38]. We have found that this reporter had a high activity in S2 cells (Figure 8 C). In this fragment we have identified two putative Da binding sites, between nucleotides −976 and −205 (Figure 8 A). An Electrophoretic Mobility Shift Assay was performed using a Da-GST fusion protein and the 0.7 Kb *string* minimal promoter containing the putative Da binding site. This assay showed that Da could bind to this fragment. Our results indicate that this binding was specific, because it could not be competed by oligonucleotides with a mutated binding site (Figure 8 B). Different results indicate that Da could bind to this region in physiological condictions. Thus, this 0.7 Kb *string* promoter fragment has been identified as an *in vivo* binding site for Da by Drosophila modENCODE project (Figure 5 A). We confirmed this result in the ChIP assay that we carried out to study *stg* promoter region (Figure 5 C).

We found that the activity of *luciferase* under the regulation of the 0.7 Kb *string* promoter was strongly down-regulated in S2 cells in the presence of Da (Figure 8 C).

When we deleted the two Da-binding sites contained in the 0.7 Kb *string* promoter, the activity of the mutant reporter was no longer repressed by Da, implying that the Da binding sites present in the 0.7 Kb *string* reporter are essential for its repressive activity (Figure 8 C). Surprisingly, *luciferase* expression driven by the mutant form of the reporter was less than that which is driven by the control reporter, suggesting that Da-binding sites are necessary to promote the normal activity of the *string* reporter. Although our results do not rule out the possibility that Da could form heterodimers with different factors to mediate this function, they indicate that the overexpression of Da is sufficient to initiate the repression of *string*.

The *Rep* domain of the Da protein has recently been shown to mediate the repression of Da/Twist heterodimer activity during myogenesis [40]. We investigated whether this domain was also involved in the repression of *string*. Overexpression of a truncated form of Da that lacks this domain (*sal^EPv^-Gal4 UAS-GFP/+; UAS-da-Δ -Rep*) caused the same defects in cell division as wild type Da overexpression (Figure S6 A–G) and it also repressed *string* expression (Figure S6 H–J). We therefore concluded that this domain is not involved in the regulatory effect of Da on cell growth.

## Discussion

It is widely accepted that basic helix-loop helix (bHLH) transcription factors, E proteins and their inhibitors, the Id factors, play important roles in controlling the balance between proliferation and differentiation that determines the correct proportion of differentiated cell types in the adult nervous system. In addition to their influence on nervous system precursors, these factors act as essential proliferative factors for a large variety of cell types in which they are expressed [16]–[18], [20], [49], [50]. In *Drosophila*, most epithelial cells express the sole representatives of class I and V bHLH factors found in this organism, Daughterless and Extramacrochaetae, respectively. In the present and previous studies, overexpression of *da* or the absence of *emc* in different tissues has been shown to block cell proliferation. These findings are consistent with the hypothesis that the expression of these factors defines thresholds for the differentiation of most cells, controlling the timing of differentiation and exit from the cell cycle [51]–[52].

In *Drosophila*, an evolutionarily conserved cross-interacting regulatory network that links E proteins and class V gene expression was recently identified [30]. Accordingly, changes in Emc levels alter *da* expression, and moreover, the effects of *emc* depletion on the control of cell proliferation are mediated by the upregulation of *da* expression. The present findings confirm that the elimination of *da* in *emc* mutant cells suppresses the growth defects caused by *emc*. Understanding how E proteins control cell proliferation is crucial to identify the mechanism by which *emc* regulates this process. Very little is known about the role of Da in controlling cell proliferation. Our results indicate that the elimination of *emc* or the overexpression of *da* results in a greater accumulation of cells in the G2 phase. Strikingly, the growth defects observed in these mutants were almost abolished by ectopic expression of *string*. Moreover, we present different results that indicate that Da transcriptionally regulates *string* expression. Our data are consistent with a model in which *da* binds to the *stg* promoter to regulate its expression. We have defined a regulatory region upstream of the transcriptional initiation site of *stg* that drives the expression of this gene in the wing discs and that is transcriptionally regulated by Da. In this region as well as throughout *stg* promoter we have identified multiple putative Da binding sites. The results obtained with our ChIP experiments, as well as the data from the modENCODE annotation project, suggest that Da binds *in vivo* to different regions of the *stg* promoter, including the regulatory region that we have identified as required for the regulation of the *stg* expression in the discs. Considered together, these results suggest that Da exerts this function binding to different regions of *string* promoter to repress its expression. Based on these findings, we propose that the main mitotic defect provoked by the overexpression of *da* or the absence of *emc* is the transcriptional downregulation of *string*.

Given the regulatory network that exists between Emc and Da [30], changes in Emc expression can modulate the levels of Da. Thus, when the levels of Emc were reduced, the levels of Da would increase. When the levels of Da rise above a certain threshold, the expression of *string* would be reduced, and then the cells will be retained in the G2 phase. Therefore, Da levels must be kept below this threshold in order for cells to remain in the proliferative cycle. Although our data indicate that the main mitotic defect caused by the overexpression of *da*, is the transcriptional down-regulation of *string*, some of our observations also suggest that *da* might be also affecting other factors that regulate cell cycle. Thus, whereas *emc^1^* mutant cells exhibited a prolonged cell doubling time compared with control cells, alterations in other G/M regulators did not change the total duration of cell cycle [53]. A plausible explanation for this discrepancy is that the expression of different target genes involved in the control of cell cycle might be regulated by Da.

According to our model, we would expect the loss of *da* to increase *string* expression. However, neither the ectopic expression of *emc* nor the elimination of *da* induced the ectopic expression of *stg*. Although we do not fully understand the reasons for this, one possibility is that the system ensures its robustness through the existence of genetic redundancy. This redundancy may occur with other bHLH genes or another transcriptional repressor, and could ensure that cell proliferation will be precisely regulated even in the absence of one or more genes. Alternatively, it has previously been shown that different antagonistic transcriptional regulators control the expression of *stg*. For example, during eye development, it has been proposed that Pointed, an activator, and Tramtrack69, a repressor, directly regulate the transcription of *string*. The absence of the repressor was not sufficient to promote the transcription of *stg*, as a positive signal is necessary to activate its transcription [54].

The absence of *emc* blocks cell proliferation in different tissues. However, the proliferation of cells completely null for *emc* function can be recovered using the *Minute* technique [55], suggesting that while *emc* is not absolutely required for cell division, its activity is necessary for the competitive success of cells. We have shown that *string* downregulation is the main mitotic defect caused by the absence of *emc* or the overexpression of *da*. Cells homozygous for a null *stg* allele divide only once, generating clones of two cells that are eliminated by cell competition [56], [37]. Interestingly, very large clones of *string* mutant cells can be generated when the function of *string* is not completely eliminated and clones are induced using the *Minute* technique [37]. Hence, the longer cell cycle of cells in which *string* is depleted would appear to result in slower growth than that which is seen in wild-type neighbours, which subsequently outcompete the *string*-deficient cells. Accordingly, the downregulation of *string* caused by the elimination of *emc* or overexpression of *da* can produce a similar growth defect.

A large body of evidence demonstrates the involvement of Id proteins in the control of cell proliferation. This family of proteins has been extensively linked to cancer in humans and it mediates several processes that are regarded as hallmarks of cancer [57]. Id proteins trigger entry into S-phase, relieving E2F transcription from the inhibitory influence of pRB. Moreover, Ids interfere with the transcriptional activation by bHLH proteins of the cyclin-dependent kinase inhibitor p21/WAF1/CIP1. While much less is known about the role of E proteins as cell cycle regulators, our data from *Drosophila* define a new mechanism through which the E protein Da and its inhibitor, the Id orthologue *emc*, regulate cell cycle progression. In our model, *emc* is required to downregulate *da*, which in turn triggers mitosis via the transcriptional activation of a universally conserved cell cycle component, Cdc25. Since the mechanisms controlling cell cycle progression are evolutionarily conserved, it seems possible that the mechanism described here will be conserved in organisms other than *Drosophila*, and that mammalian E and Id proteins may also regulate cell proliferation. It will be important to ascertain whether E proteins repress mitosis by downregulating *cdc25* expression. Although the role of Id factors in regulating G1-S transition is well documented, our data suggest that E proteins and class V factors also influence the G2 phase of the cell cycle. If the only role of Id factors were to promote the G1-S transition, they might simply arrest cells in the next phase of the cell cycle, the G2-M transition. However, the fact that Id proteins are associated with the development of tumours suggests that these factors drive cells through the different phases of the cell cycle.

## Materials and Methods

### Drosophila genetic strains

The Gal4 *Drosophila ap-Gal4*, *en-Gal4* and *sal^EPv^-Gal4* lines were used in these experiments. We also used the Janelia Gal4 GMR_32B06, GMR_32C11 and GMR_32F08 lines. To restrict the expression of the Gal4 lines, we used *tubG80^ts^*, which inhibits *Gal4* at 25°C. We used the *UAS* lines *UAS-GFP*, *UAS-mCD8-GFP, UAS-da* (kindly provided by I. Rodriguez and N. Baker), *UAS-sc* (kindly provided by S. Campuzano), *UAS-diap I, UAS-emc* and *UAS-stg*, as well as *UAS* lines to express the interference RNAs *emc-RNAi* (VDCR 100587), and *da RNAi* (VDRC 51300). The *stg-107–112 LacZ* (chr3R: 25.107.755–25.112.777) line was kindly donated by C.S. Lopes and F. Casares.

### Generation of mosaics

Mitotic clones were generated by FLP-mediated mitotic recombination. Clones lacking *emc* were obtained by crossing *emc^1^FRT2A* with *y w hsflp/FM7; tub-Gal4-UAS-GFP/Cyo; tub-Gal80-FRT2A/TM6B*. Control clones were generated using the *FRT2A* chromosome. Clones of *emc^AP6^* were generated by crossing *y w hsflp*; *emc^AP6^ FRT80/TM6B* with *y w hsflp*; *Ubi-GFP-FRT80/TM6B* flies. Clones lacking both *emc* and *da* were generated by crossing *y w hsflp; da^3^ ck FRT40; p(da^+^)Ubi-GFP-FRT80/SM6a-TM6B* with *w; da^3^ ck FRT40; emc^AP6^ FRT80/S-T* (the *emc^AP6^* and *da^3^* alleles were kindly provided by N. Baker). The clones lacking the *E(spl)* complex and simultaneously overexpressing *UAS-da* were generated by crossing *UAS-da; E(spl)^b32.2^ FRT82* with *y w hsflp tub-Gal4-UAS-GFP; tub-Gal80-FRT82/TM6B*. The progeny of these crosses were heat-shocked at 37°C for 1 hour between 48 and 72 hours after egg laying (AEL).

Clones of cells expressing Gal4 [58] were induced 48–72 hours after egg laying by heat shock at 37°C for 12 minutes in larvae *FLP1.22*; Act5C<FRT*yellow* ^+^FRT>*Gal4* UAS-*GFP*/+ UAS-*da*.

To obtain clones expressing *UAS-da* using the Gal4/Gal80 system, we crossed *UAS-da; FRT82* flies with *y w hsflp tub-Gal4-UAS-GFP; tub-Gal80-FRT82/TM6B*. The progeny of these crosses were heat-shocked at 37°C for 1 hour between 48 and 72 hours after egg laying.

*emc^1^ Df(3L)H99 M^+^* and *emc^1^ M^+^* clones were induced by crossing *emc^1^mwh Df(3L)H99* and *emc^1^ mwh* flies by *M(3)^i55^*. The progeny of these crosses were irradiated between 48–72 hours after egg laying. Mitotic recombination was induced by X-ray in a Philips MG X-ray source operated at 100 KV, 15 mA; 2-mm A1 filter at a dose of 1000rad.

Discs were dissected and analysed 3 days after clonal induction. The clone size was quantified by counting the number of GFP-positive cells after staining with the nuclear marker TOPRO.

Cell doubling time was calculated as described previously [35].

### Mitotic index

We calculated the mitotic index as the average value of the ratio between the number of cells in mitosis (PH3-positive cells) in *sal^EPv^-Gal4* or *en-Gal4* domains and the area defined by the domains of expression these Gal4 lines in pixels (PH3-positive cells/size of *sal^EPv^-Gal4* or *en-Gal4* domains). The *sal^EPV-^Gal4* and *en-Gal4* domains of expression were defined by the expression of *UAS-GFP*. We analyzed at least 5 discs for each experiment. We only considered the wing blade and hinge territories. We measured the area using Photoshop.

All experiments were compared by Student's t-test. A p-value ≤ 0.05 was considered statistically significant.

### Immunohistochemistry

Immunostaining of imaginal wing discs was performed according to standard protocols and using the following antibodies: rabbit anti-phospho-Histone 3 (1∶1000; Cell Signalling), rabbit anti-Emc (1∶50; kindly provided by Y.N. Jan), rabbit anti-Da (1∶100; kindly provided by C. Crominller), and mouse anti-ß-Gal (Promega). Mouse anti-Wg (1∶50) and mouse anti-CycB (1∶10) were obtained from the Developmental Studies Hybridoma Bank at the University of Iowa. Phalloidin-TRITC (Sigma) and TOPRO (Invitrogen) were also used to stain cell membranes and nuclei, respectively. All secondary antibodies (Molecular Probes) were used at dilutions of 1∶200.

### Fluorescence Activated Cell Sorting (FACS)

Third instar wing imaginal discs from 30 larvae *ap-Gal4 UAS-GFP/+; emc-RNAi/+* and *ap-Gal4 UAS-GFP/+* (control discs) or *sal^EPv^-Gal4 UAS-GFP/UAS-da* and *sal^EPv^-Gal4 UAS-GFP/+* (control discs) were incubated for 30 minutes at 28°C in 300 µl of trypsin solution (trypsin-EDTA, Sigma T4299) containing 1 µl of Hoechst (Hoechst 33342, trihydrochloride trihydrate H3570, Molecular Probes) in agitation. Trypsin digestion was stopped by the addition of 200 µl of 1% foetal bovine serum (FBS, Sigma 9665) in PBS. After centrifugation at 3500 rpm at 4°C, cells were suspended in 200 µl of 1% FBS and the cell cycle profiles of GFP-positive and GFP-negative cells were quantified on a FACS Vantage 2 (Becton Dickinson). The cell cycle profile of at least 3 independent experiments for each genotype was analysed using FloJo 7.5 software, and the differences were considered statistically significant at p-value ≤ 0.05.

### *In situ* hybridization

The digoxigenin-labelled *stg* RNA probe was synthesized by Baonza and Freeman (2002) [54]. To perform the *in situ* hybridization protocol, third instar larvae were dissected in cold PBS-DEPC and fixed for 20 minutes in 4% formaldehyde, washed three times for 5 minutes in PBT (PBS-DEPC - 0.1% Tween 20), and fixed a second time for 30 minutes in 4% formaldehyde in PBT. After a further 3 washes in PBT, larvae were kept at −20°C in Hybridization Solution (HS: 50% formamide, 5× SSC, 100 mg/ml salmon sperm DNA, 50% heparin, 0.1% Tween 20). The hybridization was carried out overnight at 55°C with the probe at 1/50 dilution in HS, previously denaturalized by 10 minutes' incubation at 80°C. The discs were washed three times at 55°C in HS for 5 minutes, and washed again for several times in PBT. The discs were incubated for 2 h with an anti-digoxigenin antibody (Roche) in a 1∶4000 dilution in PBT. The colour reaction was carried out in 100 mM NaCl, 50 mM MgCl_2_, 100 mM Tris-HCl pH 9.5, 0.1% Tween 20, Nitroblue Tetrazolium Chloride (NBT) and Bromo-Chloro-Indolylphosphate (BCIP) (Roche). After colour was developed, the dissected larvae were rinsed several times in PBT and the discs were mounted in 70% glycerol. The discs were photographed with a Spot digital camera and a Zeiss Axioplan microscope.

### RNA isolation and quantitative Real-Time PCR

Total RNA was isolated from a pool of 60 imaginal wing discs of the following genotypes: *WT*, *ap-Gal4/+; UAS-emc^RNAi^* and *en-Gal4/UAS-da; tubG80^ts^/+* (maintained at 29°C for 48 h during larval development to allow Gal4 expression), using the TriPure extraction protocol (Roche). After DNAse treatment (DNA-free DNAse Treatment and Removal Reagents, Applied Biosystems), total RNA (1 µg) was used for reverse transcription employing the Superscript III First Strand Synthesis Supermix kit for qRT-PCR (Invitrogen). Quantitative PCR analysis was performed in a Cfx 384 Real-Time System (BioRad) using the following primers: 

*stg* 5′ CAGCATGGATTGCAATATCAGTA 3′ and 5′ ACGACAGCTCCTCCTGGTC 3′.

*cycB* 5′ GATGCGGCACAGAAAAGACTC 3′ and 5′ TTCTTCCAGTGGCTGTCCA 3′

To adjust the differences between cDNA samples, we studied the expression of three genes that showed constitutive expression in the wing discs; they were *act42A*, *tub84A* and *rpl32*. We chose *act42A* expression to normalize the data. *stg* and *cycB* expression in control *Wild Type* flies was considered 1 and was compared to the expression in *ap-Gal4/+; UAS-emc^RNAi^* and *en-Gal4/UAS-da; tub-Gal80^ts^* flies. Four independent experiments were performed, and the cDNA variation was compared by Student's *t*-test. A p-value ≤ 0.05 was considered statistically significant.

### Electrophoretic mobility shift assay (EMSA)

A Da-GST fusion protein was generated. *da* DNA was amplified by PCR with KOD enzyme (Novagen) using the primers 5′ CACCATGGGCGACCAGTGACGATG 3′ and 5′ CTATTGCGGAAGCTGGGCGTG 3′, using the EST LD29375 as template. The purified PCR product was cloned directionally into the pENTR/D-TOPO vector (Invitrogen). To generate the GST fusion protein, we used the LR Clonase II enzyme to introduce *da* into the pDEST 15 vector which contains a GST sequence N-terminal to the recombination sites (Invitrogen). Selected positive clones were verified by sequencing. The *da* - pDEST 15 construct was transformed into *E. coli* BL21 DE3 strain. Protein expression was induced with 1 mM IPTG for 2 h at 30°C, and soluble proteins were extracted following standard procedures. The Da-GST fusion protein was purified by affinity chromatography using a Glutathione Sepharose 4B column (GE Healthcare). Purified proteins were eluted with 10 mM Glutathione. Protein-containing fractions were concentrated using Amicon Ultra 10K columns (Millipore) and analysed in a 10% polyacrylamide gel stained with Coommasie Blue. Protein concentration was determined using Bio Rad DC protein assay.

Band-shift assays were performed using the following primers:

*Stg prom cy3 and cy5*: 5′ TAGCTCATCAGCTGATCGTGA 3′ and 3′ TCACGATCAGCTGATGAGCTA 5′;

Stg prom cy5*: 5′ TAGCTCATACTAGTATCGTGA 3′ and 5′ TCACGATACTAGTATGAGCTA 3′.

The mutated primer was generated by switching purine for pyrimidine bases. Double-stranded primers (50 ng) were incubated with 5 mg of Da-GST protein for 30 minutes at room temperature in the following 5X buffer: 50 mM HEPES (pH 7.6), 200 mM KCl, 2 mM EDTA, 5 mM MgCl_2_, 1.5 mM DTT. Samples were run in a 6% DNA Retardation Gel (Invitrogen) for 3 h at 90 V protected from the light and then analysed in a fluorescence scanner.

### Chromatin immunoprecipitation and quantitative Real-Time PCR assay

To perform this assay we followed the protocol published in [59], but on a smaller scale. For each chromatin preparation we fixed 200 mg of 0–24 h *WT* embryos in 670 µl crosslinking solution, 33 µl Formaldehyde 40% and 2 ml n-Heptane, shaking vigorously for 15 minutes. After washing steps, we performed cell lysis steps in 1 ml volume. After nuclear lysis, the 1-ml aliquots were split into 100-µl aliquots for the sonication step. This was performed on a Bioruptor NextGen (Diagenode), in 30 cycles of 30 sec ON/30 sec OFF. 50 µl of sonicated chromatin were kept to perform the chromatin quality check and the rest was liquid nitrogen-frozen and stored at −80° until use. The size distribution of the sonicated chromatin was analysed in a 1% agarose gel stained with EtBr. Chromatin fragments obtained were distributed between 1 Kb and 100 bp, with a predominance of the 500 bp fragments.

Immunoprecipitation was carried out with 200 µl of sonicated chromatin per experiment. To assess the capability of Daughterless to bind different regions of DNA, we used an anti-Da rabbit polyclonal antibody at a 1∶200 dilution, kindly provided by C. Cronmiller. All the immunoprecipitations were eluted in the final step in a 10 µl final volume.

We analysed three different DNA regions that were candidate to be Da targets. As a positive control, we amplified a fragment of the *achaete* promoter (chrX: 262.623–265.355) containing two Da binding sites. This gene has been extensively studied as a direct target of Da transcriptional activity [60]. Inside the *string* regulatory region, we studied the promoter region, located in chr3R: 25,081,039–25,081,121 (named in Figure 5 as *stg prom*). We also analysed a region included in the *stg-107–112 LacZ* line and the Janelia *GMR_32F08* line that contains an *stg* enhancer that directs its expression in the wing disc (Figure 5). This fragment was previously annotated as a Da target in the modENCODE project. It contains several putative binding sites for Da, symbolized in Figure 5. We amplified a small fragment inside this region located in chr3R: 25,111,328–25,111,401 (named in Figure 5 as *stg 25.111*). As negative control, we used the gene *CG12255* that codes for the Cuticular Protein 72Eb (chr3L: 16,358,278–16,358,337). This gene does not contain any Da putative binding site.

Quantitative PCR analysis was performed in an AB 7900HT (Applied Biosystems), using the following primers:

*ac* promoter

Fw 5′ GGTATCAGGGCCTAGGGATCC 3′

Rv 5′ GATCCTTCAGTGATGATGCTGTTG 3′

*stg* promoter

Fw 5′ CGCGCCCATTAGCTCATC 3′

Rv 5′ CGAATGTGCTGAATATGCCG 3′

*stg 25.111*

Fw 5′ GTTTGCTTTAGCGGGAAACTC 3′

Rv 5′ CGGATTGCGCAAGAACAG 3′

*CG12255*

Fw 5′ CCGTGGATGGTGTGATCC 3′

Rv 5′ TCTGGGTTTCGCCATTTG 3′

Since DNA concentration was very low in the immunoprecipitated samples, we charged 2 µl of DNA on each PCR. We also ran 5 ng of a non-immunoprecipitated sample (Input) to check the availability of the sequences and that the primers functioned correctly.

To estimate the differences in DNA content between the different immunoprecipitated samples, we compared the results obtained for the different regions in study (*achaete* and *string* regulatory regions) with the data obtained for the *cg12255*. More specifically, we subtracted the Quantification Cycles (Cq) value from the mean Cq obtained for the *CG12255* (ddCq), and then calculated the Relative Quantity of template (RQ) using the formula RQ = −2^ddCq^.

All experiments were repeated at least 3 times and the DNA variation was compared by Student's t-test. A p-value ≤ 0.05 was considered statistically significant.

### Luciferase reporter experiments

A 770 bp (−976 to −205) region upstream of the transcriptional initiation site was amplified using KOD enzyme (Novagen) by PCR using the *stg 10.5* line (kindly provided by B. Edgar) as template, using the following primers: 5′ TGGGGCTCCACACTATTTTC 3′ and 5′ GATGGTAGTCCTTGGTTTTTGG 3′. The PCR product was cloned into the *pGL2* vector downstream of the *HS43* promoter using the KpnI (5′) and XhoI (3′) restriction endonucleases (NE Biolabs). To mutate the two E-boxes present in this fragment, we used Pfu enzyme (Promega) to perform PCR using the aforementioned *stg-pGL2* construct as template to sequentially eliminate both binding sites. The PCR products were subsequently digested with the DpnI enzyme (Roche). Deletion of both E-boxes was confirmed by sequencing. The following primers were used:

Deletion of first binding site,

5′ CGCCCATTAGCTCATATCGTGATCGGCCGG 3′

5′ CCGGCCGATCACGATATGAGCTAATGGGCG 3′;

Deletion of second binding site,

5′ GGGTGCGGTTATAAAAACCTCGGCATATTCAGC 3′

5′ GCTGAATATGCCGAGGTTTTATAACCGCACCC 3′.

To overexpress Da in the S2 cells, the Da coding sequence was amplified with KOD enzyme (Novagen) by PCR using the EST LD29371 as template. The primers 5′ ATGGCGACCAGTGAC 3′ and 5′ CTATTGCGGAAGC 3′ containing the restriction endonucleases EcoRV (5′) and NotI (3′) were used. The purified PCR product was cloned into the pAC5.1 vector using T4 ligase (Promega). Subconfluent *Drosophila* S2 cells (2×10^6^) were transfected with 2 µg of total DNA by electroporation using Nucloeofector (Lonza), and different concentrations of *da*-pAC5.1 (0.25 to 1 µg) were transfected with similar repressive results. Renilla plasmid (30 ng) was co-transfected in each experiment as a control for transfection efficiency. Cells were grown after transfection for 24 h at 25°C in 10% FBS Insect Xpress media (Lonza) and the Dual-Luciferase Reporter Assay Kit (Promega) was used to develop luciferase activity. All experiments were performed at least three times and compared by Student's t-test. A p-value≤0.05 was considered statistically significant.

### Quantification of the levels of GFP

GFP reporter activity was established by quantifying GFP levels in imaginal wing discs expressing *UAS-da* and *UAS-GFP* under the control of the different Gal4 used, and comparing them with GFP expressed by the same Gal4 lines without expressing *UAS-da*. ImageJ was used to measure the intensity of GFP in the wing blade. For each experiment, at least 9 wing discs were quantified using the measurement function. We calculated the average levels of GFP activity. We used the mean grey value to define the intensity of the GFP. In all cases, the images of the discs were obtained in a confocal microscope using the same settings. All experiments were compared by Student's t-test. A p-value≤0.05 was considered statistically significant

## Supporting Information

Figure S1 Cell death was not the primary cause for the phenotypes produced by the loss of function alleles of *emc* or the overexpression of *da*. (A) Control wing, *emc^1^ M^+^* and *emc^1^ DefH99 M^+^*clones, marked with *mwh* (clones are out lined in red). Veins 2 and 3 are indicated in each panel. The *emc^1^ M^+^* clone provoked the fusion of the vein 2 and the wing margin. Note that this effect was also caused by *emc^1^ DefH99 M^+^*clones. (B–D) Adult wings of genotypes: *sal^EPv^-Gal4 UAS-GFP/+* (B), *sal^EPv^-Gal4 UAS-GFP UAS-da/+* (C), and *sal^EPv^-Gal4 UAS-GFP UAS-da/UAS-diap I* (D). Note that co-overexpression of *UAS-da* and *UAS- diap I* gives the same degree of wing size reduction as *UAS-da* alone. (E–G) Wing imaginal discs of the same genotypes described in (B–D). The reduction in the number of PH3 positive cells observed when *da* was overexpressed (in red, F) was not restored by the overexpression of *diap I* (G).
(TIF)

Figure S2 *emc* and *da* regulatory loop is conserved in the Drosophila wing. (A–C) Third instar imaginal wing discs containing Control *WT* (A), *emc^AP6^* (B), and *da^3^; emc^AP6^* (C) clones marked by the absence of GFP in green. The discs are stained with anti-Wingless in red. Control twin clones were marked with double GFP. Clones of *emc^AP6^* cells do not grow in wing discs, whereas *da^3^*; *emc^AP6^* double mutant clones achieved a relatively normal size (compare C to B), as previously reported by Bhattacharya and Baker (2001) in the eye disc. (D–G) Adult wings of genotypes: *sal^EPv^-Gal4 UAS-GFP/+* (D), *sal^EPv^-Gal4 UAS-GFP/+; UAS-emc/+* (E), *sal^EPv^-Gal4 UAS-GFP/UAS-da* (F), and *sal^EPv^-Gal4 UAS-GFP UAS-da/+; UAS-emc/+* (G). The over-expression of *emc* strongly rescued the *da* overexpression phenotype (compare G with F). (H–K) Third instar imaginal wing discs of the same genotypes described in (D–G). When *UAS-emc* and *UAS-da* were simultaneously overexpressed, the defects on cell proliferation (caused by the overexpression of *UAS-da)* were strongly restored, compare K to J. Note that in discs over-expressing *UAS-da* and *UAS- emc*, we observe more mitosis (marked with Phospho-Histone 3 in red) than in discs over-expressing *UAS-da* alone (compare J with K).
(TIF)

Figure S3 The ectopic expression of *string* rescued the defects on cell proliferation caused by a reduction of *emc*. (A–D) Adult wings of genotypes: *en-Gal4 UAS-GFP/+* (A), *en-Gal4 UAS-GFP/UAS-stg* (B), *en-Gal4 UAS-GFP/+; UAS-emc^RNAi^/UAS-GFP* (C), and *en-Gal4 UAS-GFP/UAS-stg; UAS-emc^RNAi^* (D). Note that the vein fusion phenotype observed when *emc^RNAi^* was expressed in the posterior compartment was completely recovered by *stg* over-expression (compare C with D). (E–G) *en-Gal4 UAS-GFP/+*(E), *en-Gal4 UAS-GFP/+; UAS-emc^RNAi^/+*(F), and *en-Gal4 UAS-GFP/UAS-stg; UAS-emc^RNAi^/+* (G) third instar wing discs stained for Phospho-Histone-3 (PH3) (in red). (H) Quantitative analysis of the number of PH3 positive cells in the posterior compartment of the above-mentioned genotypes. The mitotic defects caused by lack of *emc* were completely recovered by *stg* overexpression. The # p-value<0.05 was established comparing *en-Gal4 UAS-GFP/+; UAS-emc^RNAi^/+* data with *en-Gal4 UAS-GFP/+* data. The * p-value<0.05 was determined comparing *en-Gal4 UAS-GFP; UAS-emc^RNAi^/UAS-stg* results with *en-Gal4 UAS-GFP/+; UAS-emc^RNAi^/+*. In all the cases we analysed 10 discs.
(TIF)

Figure S4 *da* down-regulation was not sufficient to increase *string* expression. (A, B) *ap-Gal4/+* (A), and *ap-Gal4/+; UAS-da^RNAi^/+* (B) wing imaginal discs stained with anti-Da. Da expression was eliminated in the dorsal compartment of discs over-expressing *UAS-da^RNAi^* under the control of *ap-Gal4* (compare B to A). (C, D) *In situ* hybridization against *string* mRNA in third instar wing imaginal discs of larvae *ap-Gal4/+* (C) and *ap-Gal4/+; UAS-da^RNAi^/+* (D). The D/V boundary is indicated with a white dotted line. *string* transcription was not altered when the expression of Da was reduced (compare D to C). (E) Quantitative Real-Time PCR of cDNA from imaginal wing discs of the genotypes *ap-Gal4/+* and *ap-Gal4/+; UAS-da^RNAi^/+*. No changes in *string* mRNA levels were observed when *da* levels were reduced. (F, F′) Wing imaginal discs of genotype *en-Gal4 UAS-GFP/stg-107–112 LacZ; UAS-da^RNAi^/+*, stained with anti- ß-Gal antibody (in red in F, and grey in F′). The expression of the reporter was not affected by the depletion of *da* in the posterior compartment.
(TIF)

Figure S5 The pattern of expression of the *stg*-*107–112 LacZ* reporter is similar to the pattern of expression of an *emc-GFP* reporter. (A–A″) e*mc-GFP/stg-107–112-stg LacZ* third instar imaginal wing discs stained with anti- ß-Gal antibody (in red in A, and grey in A″). The pattern of expression of *emc* is shown in green in A and grey in A′. (B) *In situ* hybridization against *string* mRNA in third instar wing discs.
(TIF)

Figure S6 Da “Rep domain” is not involved in *string* repression. (A–C) *sal^EPv^-Gal4 UAS-GFP/+* (A), *sal^EPv^-Gal4 UAS-GFP/UAS-da* (B), and *sal^EPv^-Gal4 UAS-GFP/UAS-da-Δ -Rep* (C) adult wings. Note that over-expression of a mutated form of *da* (*UAS-da-Δ -Rep*) gives the same phenotype as the over-expression of a wild type form of *da* (compare B to C). (D–F) *sal^EPv^-Gal4 UAS-GFP/+* (D), *sal^EPv^-Gal4 UAS-GFP/UAS-da* (E), and *sal^EPv^-Gal4 UAS-GFP/UAS-da-Δ -Rep* (F) third instar wing discs stained for Phospho-Histone-3 (PH3) (in red). (G) Quantitative analysis of the number of PH3 positive cells in the *sal^EPv^* area of the above-mentioned genotypes. The mitotic defects observed when a wild type form of *da* was over-expressed were similar to those caused when the “Rep domain” was ablated (*** p-value<0,001 were calculated comparing the results of *sal^EPv^-Gal4/UAS-da*, and *sal^EPv^-Gal4/+; UAS-da-Δ -Rep/+* data with *sal^EPv^-Gal4 UAS-GFP/+* results). (H–J) *In situ* hybridization against *string* mRNA in *sal^EPv^-Gal4/+* (H), *sal^EPv^-Gal4/UAS-da* (I), and *sal^EPv^-Gal4/+; UAS-da-Δ -Rep/+* third instar wing imaginal discs. *sal^EPv^-Gal4* presumptive area was marked with a white dotted line. Note that *stg* expression was reduced in the *sal^EPv^* area when the wild type or the mutated forms of *da* (*UAS-da-Δ -Rep*) were over-expressed (compare I with J).
(TIF)
