# Supplementary figures and images for: The bHLH Factors Extramacrochaetae and Daughterless Control Cell Cycle in Drosophila Imaginal Discs through the Transcriptional Regulation of the cdc25 Phosphatase string
Source: PLoS Genet. 2014 Mar 20;10(3):e1004233. doi: 10.1371/journal.pgen.1004233 (PMC3961188; doi:10.1371/journal.pgen.1004233)

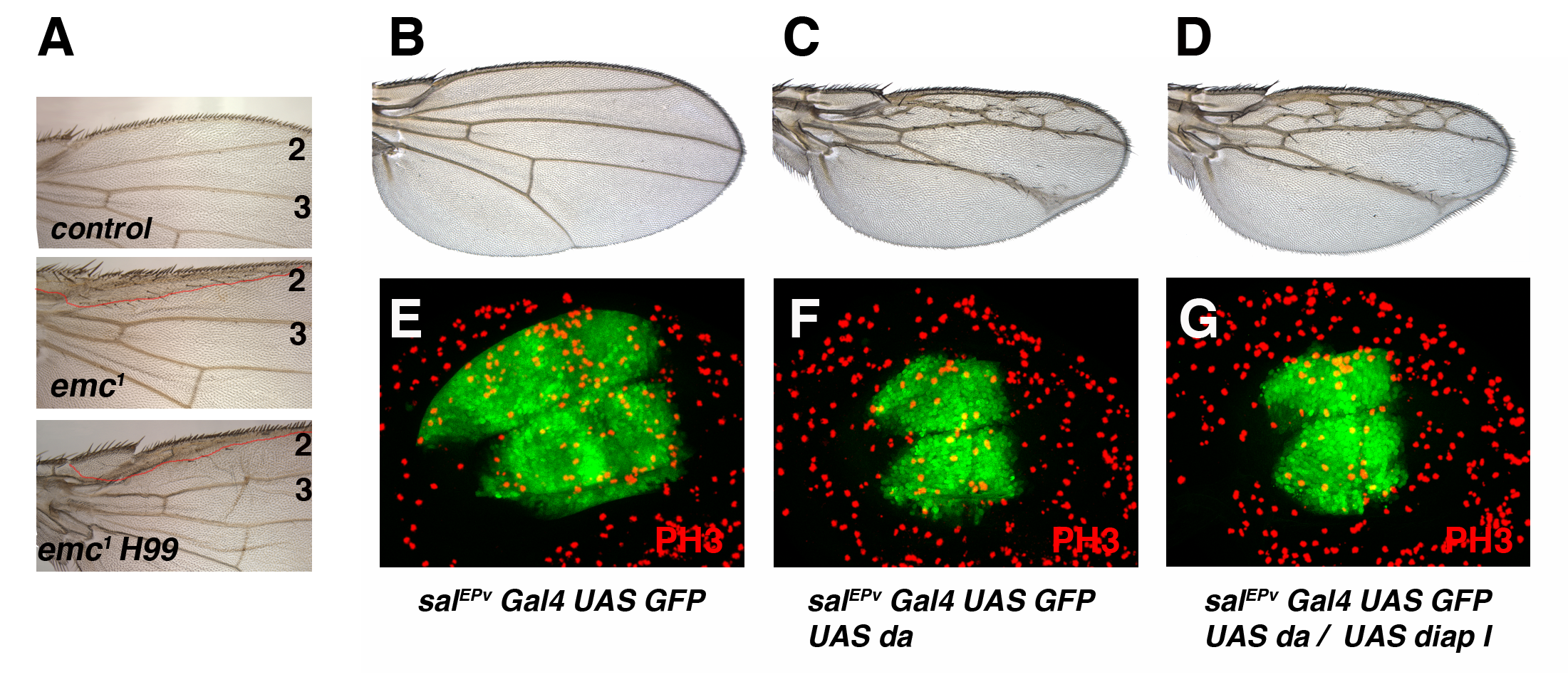

Supplement: Figure S1 — Cell death was not the primary cause for the phenotypes produced by the loss of function alleles of emc or the overexpression of da. (A) Control wing, emc1 M+ and emc1 DefH99 M+clones, marked with mwh (clones are out lined in red). Veins 2 and 3 are indicated in each panel. The emc1 M+ clone provoked the fusion of the vein 2 and the wing margin. Note that this effect was also caused by emc1 DefH99 M+clones. (B–D) Adult wings of genotypes: salEPv-Gal4 UAS-GFP/+ (B), salEPv-Gal4 UAS-GFP UAS-da/+ (C), and salEPv-Gal4 UAS-GFP UAS-da/UAS-diap I (D). Note that co-overexpression of UAS-da and UAS- diap I gives the same degree of wing size reduction as UAS-da alone. (E–G) Wing imaginal discs of the same genotypes described in (B–D). The reduction in the number of PH3 positive cells observed when da was overexpressed (in red, F) was not restored by the overexpression of diap I (G). (TIF) [file pgen.1004233.s001.tif]

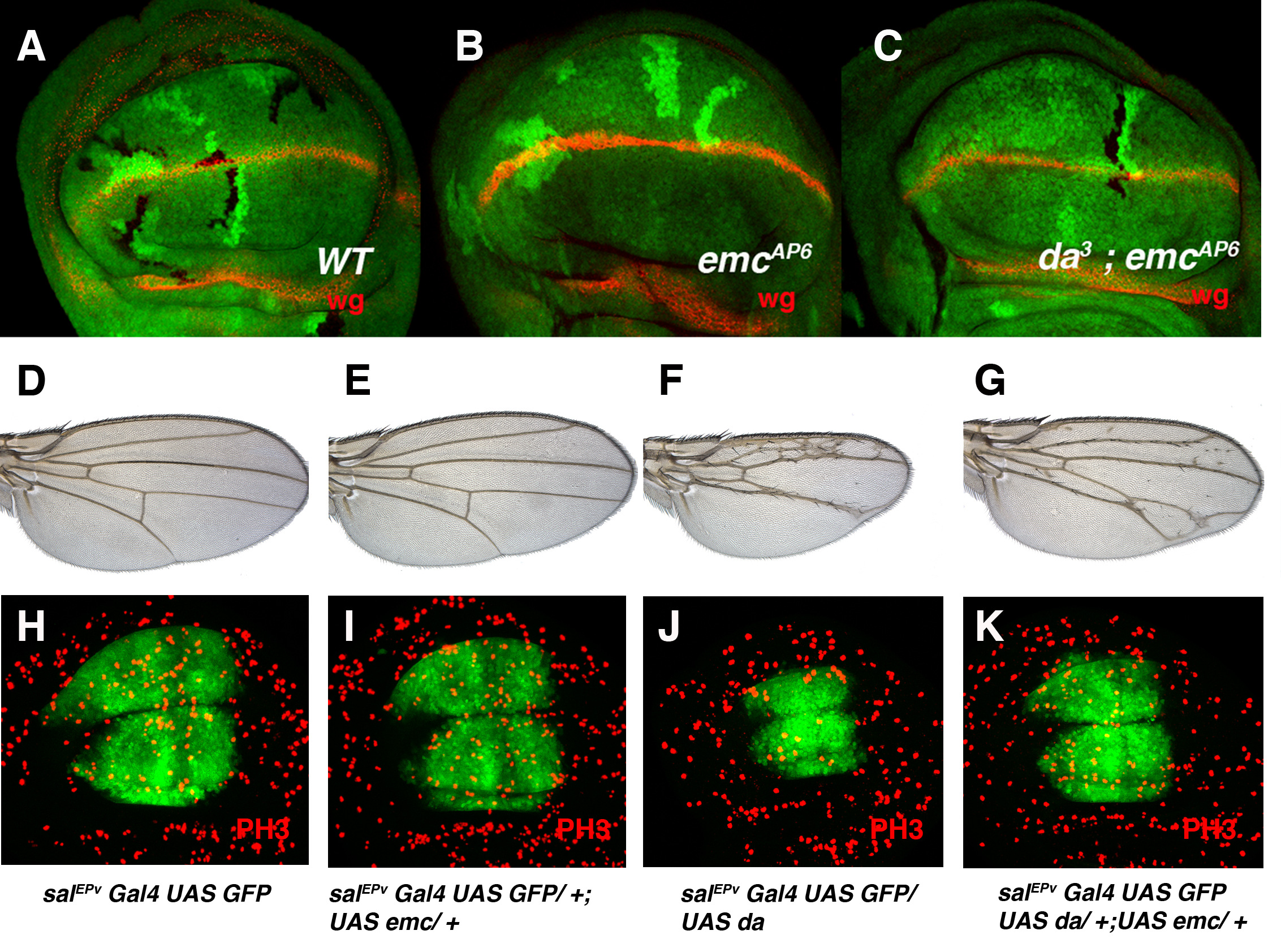

Supplement: Figure S2 — emc and da regulatory loop is conserved in the Drosophila wing. (A–C) Third instar imaginal wing discs containing Control WT (A), emcAP6 (B), and da3; emcAP6 (C) clones marked by the absence of GFP in green. The discs are stained with anti-Wingless in red. Control twin clones were marked with double GFP. Clones of emcAP6 cells do not grow in wing discs, whereas da3; emcAP6 double mutant clones achieved a relatively normal size (compare C to B), as previously reported by Bhattacharya and Baker (2001) in the eye disc. (D–G) Adult wings of genotypes: salEPv-Gal4 UAS-GFP/+ (D), salEPv-Gal4 UAS-GFP/+; UAS-emc/+ (E), salEPv-Gal4 UAS-GFP/UAS-da (F), and salEPv-Gal4 UAS-GFP UAS-da/+; UAS-emc/+ (G). The over-expression of emc strongly rescued the da overexpression phenotype (compare G with F). (H–K) Third instar imaginal wing discs of the same genotypes described in (D–G). When UAS-emc and UAS-da were simultaneously overexpressed, the defects on cell proliferation (caused by the overexpression of UAS-da) were strongly restored, compare K to J. Note that in discs over-expressing UAS-da and UAS- emc, we observe more mitosis (marked with Phospho-Histone 3 in red) than in discs over-expressing UAS-da alone (compare J with K). (TIF) [file pgen.1004233.s002.tif]

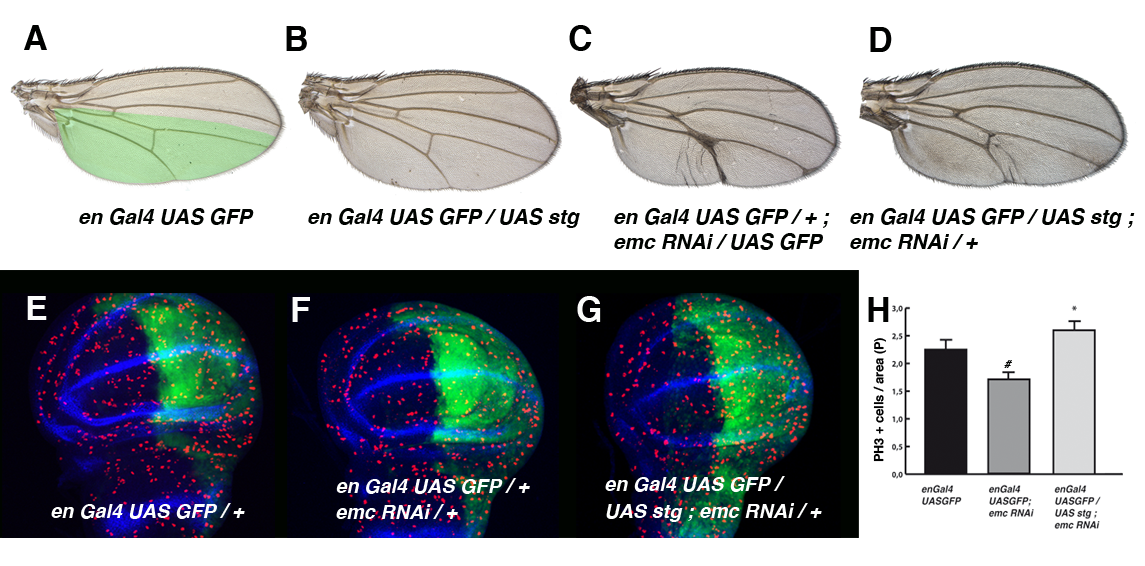

Supplement: Figure S3 — The ectopic expression of string rescued the defects on cell proliferation caused by a reduction of emc. (A–D) Adult wings of genotypes: en-Gal4 UAS-GFP/+ (A), en-Gal4 UAS-GFP/UAS-stg (B), en-Gal4 UAS-GFP/+; UAS-emcRNAi/UAS-GFP (C), and en-Gal4 UAS-GFP/UAS-stg; UAS-emcRNAi (D). Note that the vein fusion phenotype observed when emcRNAi was expressed in the posterior compartment was completely recovered by stg over-expression (compare C with D). (E–G) en-Gal4 UAS-GFP/+(E), en-Gal4 UAS-GFP/+; UAS-emcRNAi/+(F), and en-Gal4 UAS-GFP/UAS-stg; UAS-emcRNAi/+ (G) third instar wing discs stained for Phospho-Histone-3 (PH3) (in red). (H) Quantitative analysis of the number of PH3 positive cells in the posterior compartment of the above-mentioned genotypes. The mitotic defects caused by lack of emc were completely recovered by stg overexpression. The # p-value<0.05 was established comparing en-Gal4 UAS-GFP/+; UAS-emcRNAi/+ data with en-Gal4 UAS-GFP/+ data. The * p-value<0.05 was determined comparing en-Gal4 UAS-GFP; UAS-emcRNAi/UAS-stg results with en-Gal4 UAS-GFP/+; UAS-emcRNAi/+. In all the cases we analysed 10 discs. (TIF) [file pgen.1004233.s003.tif]

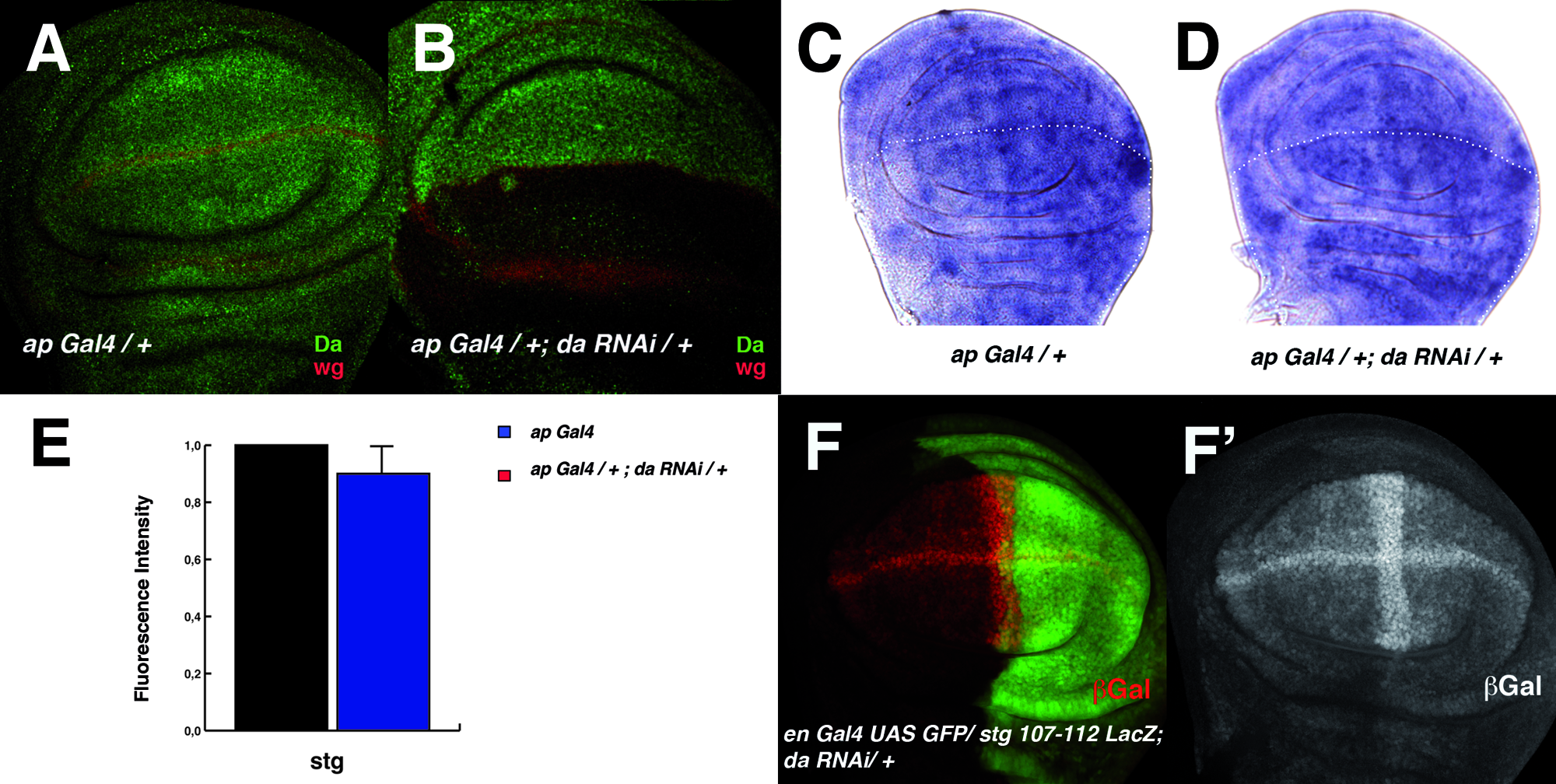

Supplement: Figure S4 — da down-regulation was not sufficient to increase string expression. (A, B) ap-Gal4/+ (A), and ap-Gal4/+; UAS-daRNAi/+ (B) wing imaginal discs stained with anti-Da. Da expression was eliminated in the dorsal compartment of discs over-expressing UAS-daRNAi under the control of ap-Gal4 (compare B to A). (C, D) In situ hybridization against string mRNA in third instar wing imaginal discs of larvae ap-Gal4/+ (C) and ap-Gal4/+; UAS-daRNAi/+ (D). The D/V boundary is indicated with a white dotted line. string transcription was not altered when the expression of Da was reduced (compare D to C). (E) Quantitative Real-Time PCR of cDNA from imaginal wing discs of the genotypes ap-Gal4/+ and ap-Gal4/+; UAS-daRNAi/+. No changes in string mRNA levels were observed when da levels were reduced. (F, F′) Wing imaginal discs of genotype en-Gal4 UAS-GFP/stg-107–112 LacZ; UAS-daRNAi/+, stained with anti- ß-Gal antibody (in red in F, and grey in F′). The expression of the reporter was not affected by the depletion of da in the posterior compartment. (TIF) [file pgen.1004233.s004.tif]

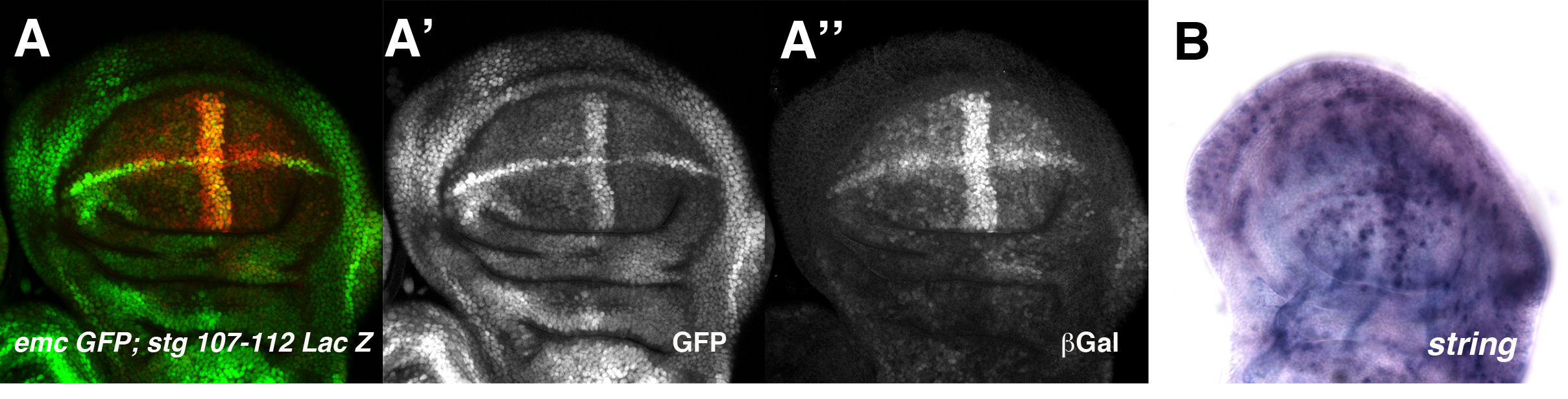

Supplement: Figure S5 — The pattern of expression of the stg-107–112 LacZ reporter is similar to the pattern of expression of an emc-GFP reporter. (A–A″) emc-GFP/stg-107–112-stg LacZ third instar imaginal wing discs stained with anti- ß-Gal antibody (in red in A, and grey in A″). The pattern of expression of emc is shown in green in A and grey in A′. (B) In situ hybridization against string mRNA in third instar wing discs. (TIF) [file pgen.1004233.s005.tif]

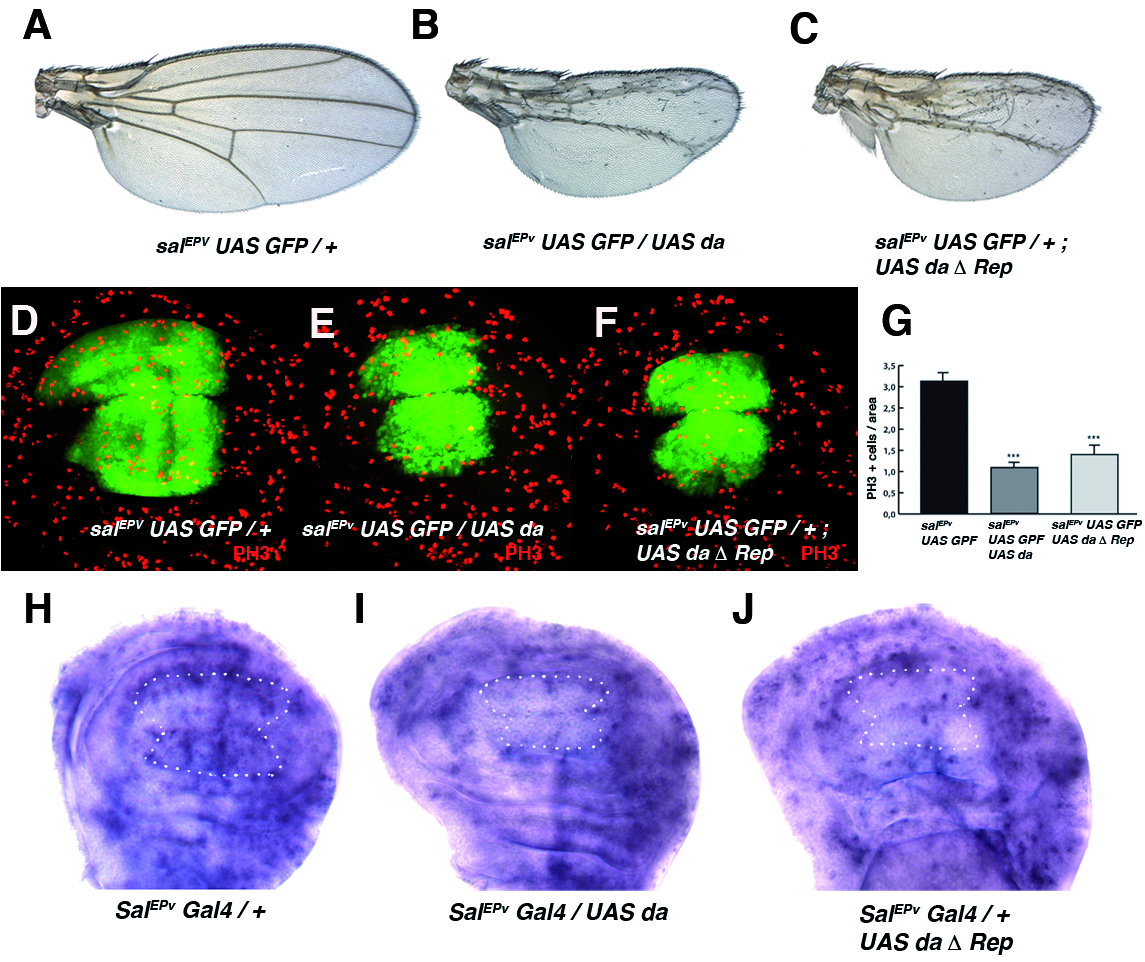

Supplement: Figure S6 — Da “Rep domain” is not involved in string repression. (A–C) salEPv-Gal4 UAS-GFP/+ (A), salEPv-Gal4 UAS-GFP/UAS-da (B), and salEPv-Gal4 UAS-GFP/UAS-da-Δ -Rep (C) adult wings. Note that over-expression of a mutated form of da (UAS-da-Δ -Rep) gives the same phenotype as the over-expression of a wild type form of da (compare B to C). (D–F) salEPv-Gal4 UAS-GFP/+ (D), salEPv-Gal4 UAS-GFP/UAS-da (E), and salEPv-Gal4 UAS-GFP/UAS-da-Δ -Rep (F) third instar wing discs stained for Phospho-Histone-3 (PH3) (in red). (G) Quantitative analysis of the number of PH3 positive cells in the salEPv area of the above-mentioned genotypes. The mitotic defects observed when a wild type form of da was over-expressed were similar to those caused when the “Rep domain” was ablated (*** p-value<0,001 were calculated comparing the results of salEPv-Gal4/UAS-da, and salEPv-Gal4/+; UAS-da-Δ -Rep/+ data with salEPv-Gal4 UAS-GFP/+ results). (H–J) In situ hybridization against string mRNA in salEPv-Gal4/+ (H), salEPv-Gal4/UAS-da (I), and salEPv-Gal4/+; UAS-da-Δ -Rep/+ third instar wing imaginal discs. salEPv-Gal4 presumptive area was marked with a white dotted line. Note that stg expression was reduced in the salEPv area when the wild type or the mutated forms of da (UAS-da-Δ -Rep) were over-expressed (compare I with J). (TIF) [file pgen.1004233.s006.tif]
